# Twelve complete chloroplast genomes of wild peanuts: great genetic resources and a better understanding of *Arachis* phylogeny

**DOI:** 10.1186/s12870-019-2121-3

**Published:** 2019-11-19

**Authors:** Juan Wang, Yuan Li, Chunjuan Li, Caixia Yan, Xiaobo Zhao, Cuiling Yuan, Quanxi Sun, Chengren Shi, Shihua Shan

**Affiliations:** 1 Shandong Peanut Research Institute, Qingdao, China; 2 Computational Biology and Biological Physics, Astronomy and Theoretical Physics, Lund University, Lund, Sweden

**Keywords:** *Arachis*, Peanut, Chloroplast genome, Phylogeny, SNDs, SSRs, Genome type, Genetic variation

## Abstract

**Background:**

The cultivated peanut (*Arachis hypogaea*) is one of the most important oilseed crops worldwide, however, its improvement is restricted by its narrow genetic base. The highly variable wild peanut species, especially within Sect. *Arachis*, may serve as a rich genetic source of favorable alleles to peanut improvement; Sect. *Arachis* is the biggest taxonomic section within genus *Arachis* and its members also include the cultivated peanut. In order to make good use of these wild resources, the genetic bases and the relationships of the *Arachis* species need first to be better understood.

**Results:**

Here, in this study, we have sequenced and/or assembled twelve *Arachis* complete chloroplast (cp) genomes (eleven from Sect. *Arachis*). These cp genome sequences enriched the published *Arachis* cp genome data. From the twelve acquired cp genomes, substantial genetic variation (1368 SNDs, 311 indels) has been identified, which, together with 69 SSR loci that have been identified from the same data set, will provide powerful tools for future explorations. Phylogenetic analyses in our study have grouped the Sect. *Arachis* species into two major lineages (I & II), this result together with reports from many earlier studies show that lineage II is dominated by AA genome species that are mostly perennial, while lineage I includes species that have more diverse genome types and are mostly annual/biennial. Moreover, the cultivated peanuts and *A*. *monticola* that are the only tetraploid (AABB) species within *Arachis* are nested within the AA genome species-dominated lineage, this result together with the maternal inheritance of chloroplast indicate a maternal origin of the two tetraploid species from an AA genome species.

**Conclusion:**

In summary, we have acquired sequences of twelve complete *Arachis* cp genomes, which have not only helped us better understand how the cultivated peanut and its close wild relatives are related, but also provided us with rich genetic resources that may hold great potentials for future peanut breeding.

## Background

The genus *Arachis* L. has gained significant research interest due to one of its member species, the cultivated peanut (*A. hypogaea* L.) that is an economically important oilseed crop worldwide [1, 2]. *Arachis* species are endemic to South America, and it is from there the cultivated peanut has been spread out and planted in many different parts of the world that are far from its cradleland, among which China represents the largest producer today [1–3].

Morphologically, two subspecies have been identified within *A. hypogaea*, which can be further sorted into six botanical varieties [4]. Although phenotypically variable, *A. hypogaea* has a relatively low level of genetic variation comparing to its wild relatives [5–10], which may be due to domestication, and a possibly recent and single polyploidization origin as well as the ploidy difference between the cultivated and wild peanut [1, 2, 11, 12]. The limited genetic variation of *A. hypogaea* restrains the further improvement of this crop, especially from the perspective of resistance to diseases and pests [1, 2]. Luckily, interspecific hybridization between the *Arachis* species is possible especially with the help of modern technology and there is a rich source of genetic variation among the closely related wild relatives within the *Arachis* genus that may be very useful for broadening the genetic basis of the cultivated peanut [2, 4, 12–15].

So far, over 80 species have been identified within the *Arachis* genus [2], which were arranged into nine taxonomic sections (including Sect. *Arachis*, Sect. *Erectoides* and Sect. *Procumbentes*) by Krapovickas and Gregory [16]; the cultivated peanut belongs to Sect. *Arachis*. Many useful resistances to a number of diseases (e.g. early leaf spot, late leaf spot, peanut rust and rosette disease) and pests (such as nematodes, armyworm and corn earworm) that can cause serious yield loss in the cultivated peanut have been identified from the wild Sect. *Arachis* species [2]. For example, accessions of *A*. *duranensis* Krapov. & W.C. Gregory and *A. cardenasii* Krapov. & W.C. Gregory that belong to Sect. *Arachis* have been found to be resistant to twelve or more different diseases/pests, representing two of the richest sources of novel resistant alleles for cultivated peanut [2, 17]. To more efficiently make use of these rich genetic resources for peanut breeding, a better understanding of the genomes and phylogenetic relationships of the species within *Arachis* is a prerequisite.

As mentioned above, there are nine taxonomic sections within the genus *Arachis* that are arranged based on morphology, crossability, cytogenetics, hybrid viability and geographic distribution [2, 4, 16, 18, 19], however, this grouping has been challenged by molecular phylogenetic studies [20, 21]. Among the nine *Arachis* sections, Sect. *Arachis* that includes the cultivated peanut is the largest, the most diverse and the most derived one: it includes more than one third of the *Arachis* species, and harbors both annual and perennial species that may also differ in chromosome number, ploidy level and genome type [1, 4]. Many attempts to infer the phylogenetic relationship among *Arachis* species have been made, though incongruence between markers and studies is very common [8, 10, 11, 14, 20, 22–26]. The rapidly developing high-throughput sequencing technology may provide us with a good chance to make use of the chloroplast (cp) genome data for helping improve the situation [27], due to several advantages of the cp genome data in phylogenetic analyses. First, cp genome data harbors many different gene loci and non-coding regions that contain relatively large amount of DNA sequence information, this would not only boost the resolving power of phylogenetic inference, but also dramatically reduce stochastic errors that are associated with limited sequence information of single genes in traditional phylogeny construction [28, 29]. Second, as being maternally inherited, cp genome in *Arachis* may provide the best tool for inferring the maternal origin of the cultivated peanut. Third, the haploid nature of the cp genome severely restrains the occurrence of non-homologous recombination events, which will make cp genome suffer less from recombination in phylogenetic analysis [30, 31]. Finally, the cp genome has a relatively small size compared to nuclear genome, which means that it’s relatively cheap and easy to sequence and analyze [27].

Apart from the advantages in phylogenetic analysis as mentioned above, the cp genome is also useful for developing DNA barcodes that can be helpful, for example, in distinguishing taxa, as well as for cp genetic engineering that transfers foreign genes into cp genomes [27]. Comparing to nuclear transgenic plants, chloroplast genetic engineering has several advantages, including a high level of transgene expression and no escape of transgenes through pollen [27]. Now there are already plenty of successful cases of cp genetic engineering that have been performed [27]. Moreover, cp genes have also been found to possibly contribute to host plants’ resistance to environmental stresses [32, 33]. Although the cp genome is very useful, there are, however, still a very limited number of cp genomes available for the *Arachis* species so far. The first two *Arachis* complete cp genomes that have been sequenced are from the cultivated peanut, *A*. *hypogeae*, and were reported rather recently by Schwarz et al. [34] and Prabhudas et al. [35]. After that, Yin et al. [21] acquired the complete cp genome sequences from seven different *Arachis* species including the domesticated peanut, while Wang et al. [36] explored the complete cp genome sequences of four *A*. *hypogeae* botanical varieties. To sum it up, there are only thirteen *Arachis* complelete cp genomes from seven different species that have been sequenced so far.

In this study, we have assembled a total of twelve complete cp genomes of *Arachis* species, among which eleven are from Sect. *Arachis* while the last one is from Sect. *Erectoides*, and these data represent a rich source of genetic variation that may hold great potential for peanut improvement. These sequences together with earlier published *Arachis* cp genome data have helped us better understand the *Arachis* cp genomes and the phylogenetic relationships among species within and among *Arachis* sections, as well as give more information about the wild maternal origin of the cultivated peanut.

## Results

### Basic characteristics of the acquired *Arachis* chloroplast genomes

**Fig. 1:**
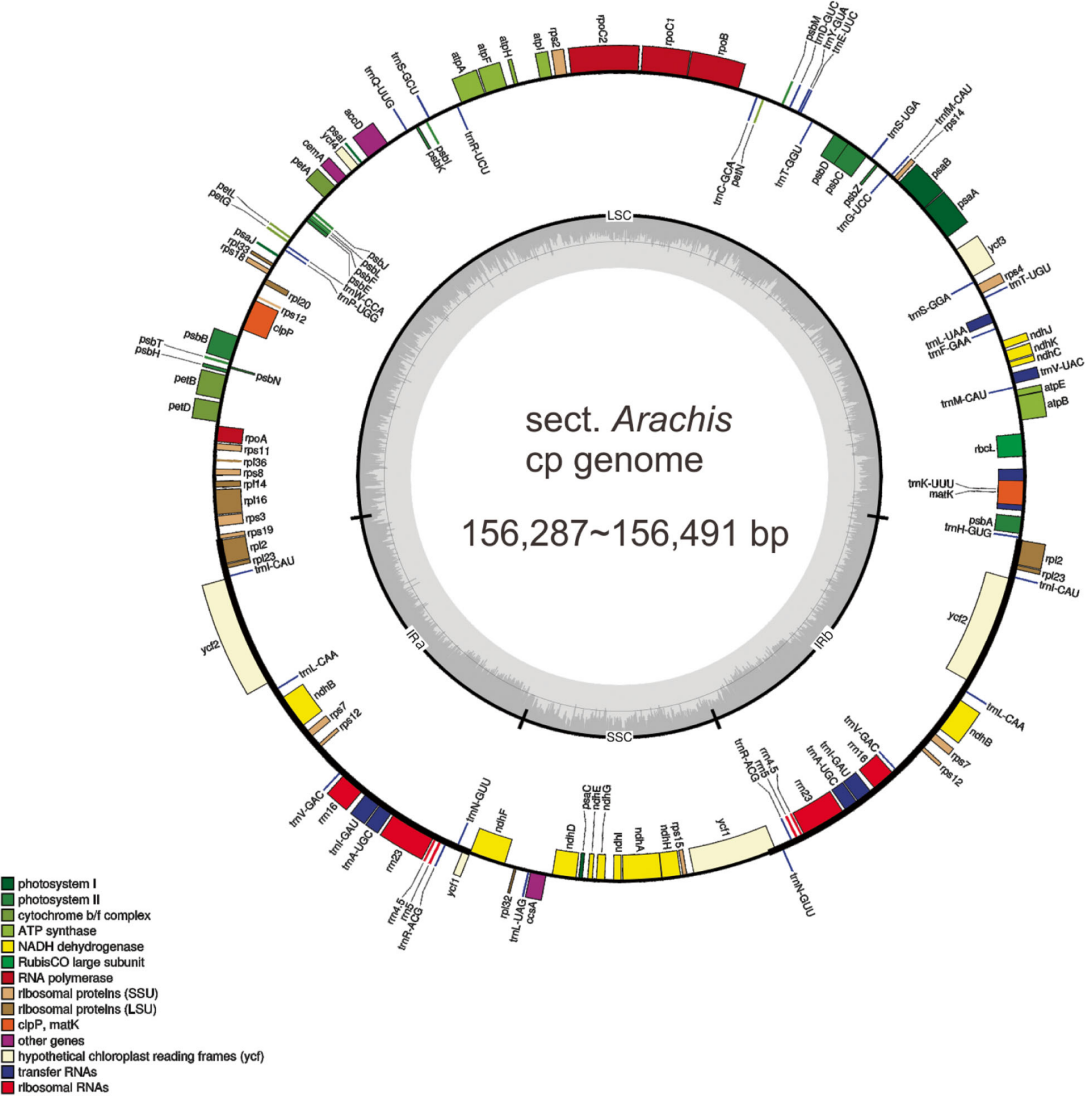
Map of the Sect. *Arachis* chloroplast genomes. Genes shown outside the outer circle are transcribed clockwise and those inside are transcribed counterclockwise. Genes belonging to different functional groups are color-coded. Gray area in the inner circle indicates the GC content of the chloroplast genome. The four regions of a chloroplast genome are also indicated in the inner circle: the two inverted repeat regions (IRa and IRb) are separated by small (SSC) and large (LSC) single copy regions

**Fig. 2:**
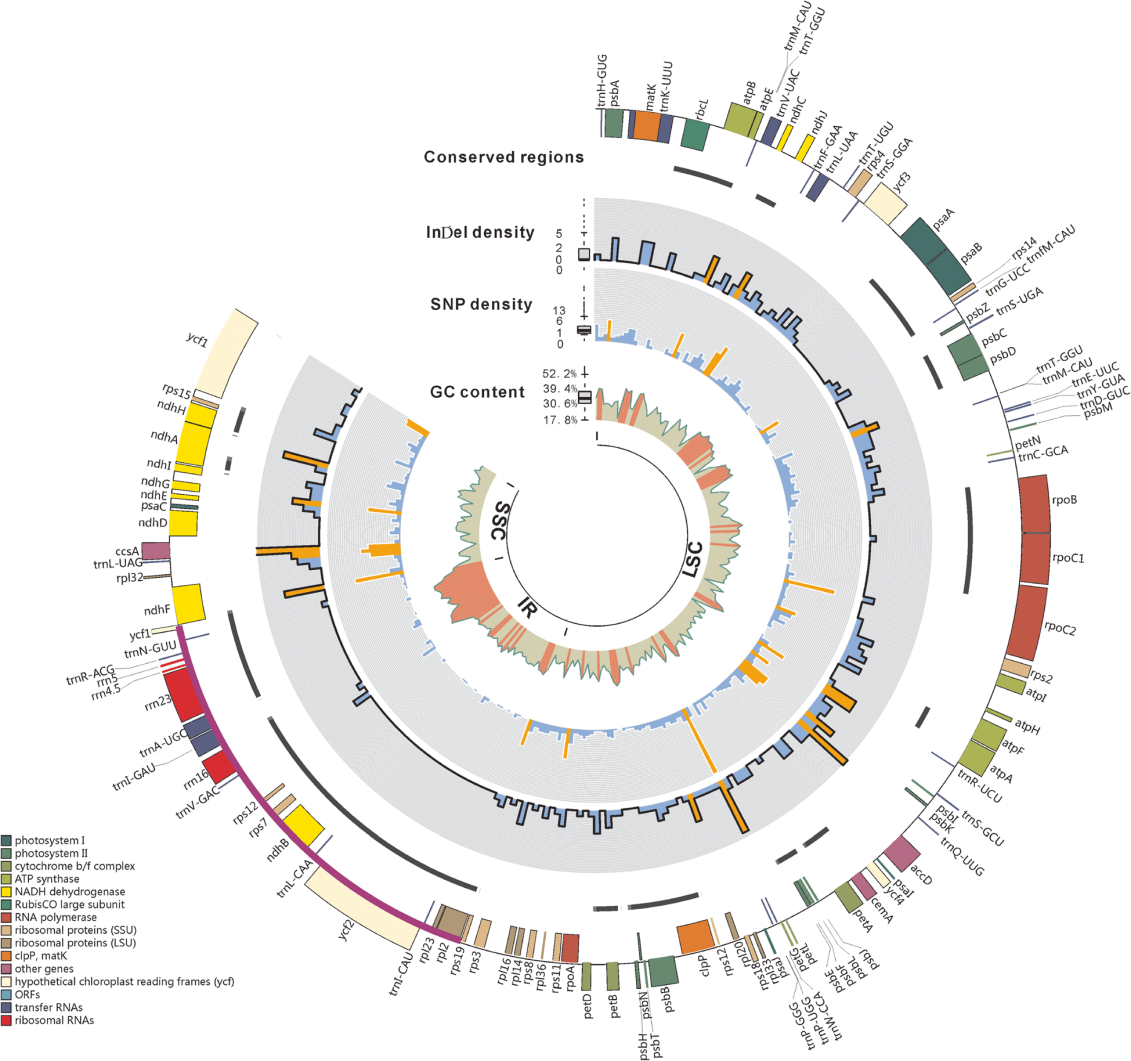
Circos plot showing basic characteristics of the twelve *Arachis* cp genomes acquired in this study. The GC content, the SND density, the indel density, and the conserved regions are shown from the inner to outer rings. The outermost rectangles are cp genes belonging to different functional groups that are color-coded. The high-density areas are highlighted in orange for GC content (> 53.06%), SNDs (> 8.39%) and indels (> 5.34%). Boxplots for the GC contents, the SND and indel densities have also been shown, with bars representing the median, the bottom and top of the boxes representing, respectively, the 25 and 75% percentiles, and whiskers extending out to 1.5 times the interquartile range. Dots are outliers

A total of twelve *Arachis* species that belong to Sect. *Arachis (A. monticola* Krapov. & Rigoni [GRIN accession number: PI-219824], *A. duranensis* [PI-263133], *A. stenosperma* Krapov. & W.C. Gregory [PI-338280], *A. batizocoi* Krapov. & W.C. Gregory [PI-298639], *A. cardenasii* [PI-262141], *A. helodes* Martius ex Krapov. & Rigoni [Collector no.: Manso 588], *A. correntina* (Burkart) Krapov. & W.C. Gregory [PI-331192]*, A. hoehnei* Krapov. & W.C. Gregory [Collector no.: KG30006]*, A. chacoensis* Krapov. & W.C. Gregory (now known as *A. diogoi* Hoehne) [PI-276235] and *A. villosa* Benth. [PI-210555], *A. ipaënsis* Krapov. & W.C. Gregory [37]) and Sect. *Erectoides (A. paraguariensis ssp. paraguariensis* Chodat & Hassl. [PI-331187]) have their cp genomes sequenced and/or assembled in the present study (Table 1). In comparison to nuclear genome, cp genomes of land plants have highly-conserved circular DNA molecules with two inverted repeat (IR) regions (IRa and IRb) (identical but in opposite orientations) that were separated by small (SSC) and large (LSC) single copy (SC) regions [38]. The twelve cp genomes assembled within this study had this typical quadripartite structure (Figs. 1 and 2), and with total lengths varying from 156,287 bp (*A. stenosperma*) to 156,491 bp (*A. cardenasii*) (Additional file 1), which were similar to those earlier published *Arachis* cp genomes [21, 34–36]. The four different genome regions ranged in size from 85,830 bp (*A. duranensis*) to 85,980 bp (*A. villosa*) for LSC, from 18,796 bp (*A. correntina* and *A. monticola*) to 18,942 bp (*A. cardenasii*) for SSC and from 25,776 bp (*A. ipaënsis*) to 25,842 bp (*A. batizocoi*) for IRa/b (Additional file 1).

**Table 1:** The *Arachis* species that have been analyzed in the present study

Species	Genome type	Life History Strategy	Ploidy	Collection site
Sect. *Arachis*				
Domesticated peanut varieties				
*A. hypogaea* var. *hypogaea*	AABB	Annual/biennial	4x	Shandong Peanut Research Institute, Shandong Academy of Agricultural Sciences, Qingdao, China (SPRI-SAAS)
*A. hypogaea* var. *hirsuta*	AABB	Annual/biennial	4x	SPRI-SAAS
*A. hypogaea* var. *fastigiata*	AABB	Annual/biennial	4x	SPRI-SAAS
*A. hypogaea* var. *vulgaris*	AABB	Annual/biennial	4x	SPRI-SAAS
Wild allotetraploid species				
*A. monticola*	AABB	Annual/biennial	4x	Nanning Branch of National Field Genbank for *Arachis* species, Guangxi Academy of Agricultural Sciences, Nanning, China (NB-NFGAS- GAAS)
Wild diploid species				
*A. batizocoi*	KK	Annual/biennial	2x	NB-NFGAS- GAAS
*A. cardenasii*	AA	Perennial	2x	NB-NFGAS- GAAS
*A. chacoensis/A. diogoi*	AA	Perennial	2x	NB-NFGAS- GAAS
*A. correntina*	AA	Perennial	2x	NB-NFGAS- GAAS
*A. duranensis*	AA	Annual/biennial	2x	NB-NFGAS- GAAS
*A. helodes*	AA	Perennial	2x	NB-NFGAS- GAAS
*A. hoehnei*	AA	Annual/biennial	2x	NB-NFGAS- GAAS
*A. stenosperma*	AA	Annual/biennial	2x	NB-NFGAS- GAAS
*A. villosa*	AA	Perennial	2x	NB-NFGAS- GAAS
*A. ipaënsis*	BB	Annual/biennial	2x	*
Sect. *Erectoides*				
*A. paraguariensis*	EE	Perennial	2x	NB-NFGAS- GAAS

* The cp genome sequence data for *A*. *ipaënsis* were downloaded from the NCBI SRA database

As with earlier reports about *Arachis* cp genomes [21, 34–36], a total of 110 unique genes were found in each of the twelve assembled cp genomes, including four ribosomal RNA (rRNA) genes, 76 protein-coding genes and 30 transfer RNA (tRNA) genes (Additional files 1 and 2). The gene order of these 110 genes was the same for all the twelve assembled cp genomes (Fig. 1), and was also in line with all published *Arachis* cp genomes so far [21, 34–36]. In addition, six of the identified tRNA genes, and eleven of the protein-coding genes contained introns (Additional file 2).

Similar to earlier studies [34, 35], the overall GC contents of the twelve assembled cp genomes were 36.3–36.4% and the GC contents were not evenly distributed among the different genome regions: IRs (42.9–43.0%) had higher GC content than LSC (33.8%) and SSC (29.9–30.3%) (Additional file 1). The high GC content of IRs was mostly due to a gene region (including the *rrn*23, *trn*A-UGC and *trn*l-GAU genes) that stands out in its GC content comparing to the rest of the cp genome (Fig. 2).

### Genetic variation and SSRs

Among the twelve cp genomes that were assembled in the present study, a total of 1368 single nucleotide divergences (SNDs) (0.87%) were identified, and most of these SNDs were distributed within the LSC region (959, constituting 1.11% of the LSC sequence), but the SSC region contained the highest proportion of SNDs (299, 1.58%), while the SND content of the IRa/b regions was the lowest (55 each, 0.21%) (Table 2, Fig. 2). The cp genome region with the highest density of SNDs was located at the intergenic spacer between the *psb*E and *pet*L genes within LSC, where 47 SNDs were found within 500 bp (Table 3).

**Table 2:** A summary of the SNDs and indels identified among the twelve acquired chloroplast genomes

		SNDs		indels	
Region	Length	Number	Proportion (%)	Number	Proportion (%)
LSC	86.197	959	1.11%	241	0.27%
SSC	18,876	299	1.58%	64	0.34%
IR	25,825	55	0.21%	6	0.02%
Complete genome	156,718	1368	0.87%	311	0.20%

**Table 3:** The genome areas identified to be rich in SNDs among the twelve acquired chloroplast genomes. Genome areas that have more than 10 SNDs per 500 bp are considered to be rich in SNDs

Genome position	Count	Region	Genomic context	Sequence content
66,000–66,500	47	LSC	*psb*E~*pet*L	spacer
44,500–45,000	34	LSC	*rpo*C2	coding
112,000–112,500	32	IRA; SSC	*trn*N-GUU~*ndh*F; *ndh*F	spacer; coding
115,000–115,500	26	SSC	*rpl*32; *rpl*32~*trn*L-UAG	coding; spacer
15,000–15,500	23	LSC	*trn*L-UAA~*trn*L-UGU; *trn*T-UGU; *trn*T-UGU~*rps*4	spacer; coding; spacer
119,500–120,000	22	SSC	*psa*C~*ndh*E*; ndh*E~*ndh*G	spacer; spacer
115,500–116,000	21	SSC	*rpl*32~*trn*L-UAG; *trn*L-UAG; *trn*L-UAG~*ccs*A	spacer; coding; spacer
53,000–53,500	20	LSC	*trn*R-UCU; *trn*R-UCU~*trn*S-GCU	coding; spacer
56,000–56,500	20	LSC	*psb*K; *psb*K~*trn*Q-UUG	coding; spacer
56,500–57,000	20	LSC	*psb*K~*trn*Q-UUG; *trn*Q-UUG; *trn*Q-UUG~*acc*D	spacer; coding; spacer
81,000–81,500	20	LSC	*rps*11~*rpl*36; *rpl*36; *rpl*36~*rps*8	spacer; coding; spacer
114,500–115,000	20	SSC	*ndh*F~*rpl*32; *rpl*32	spacer; coding
86,000–86,500	18	LSC;IRA	*rps*19; *rps*19~*rpl*2; *rpl*2	coding; spacer; coding
10,000–10,500	17	LSC	*trn*V-UAC; *trn*V-UAC~*ndh*C	coding; spacer
14,500–15,000	17	LSC	*trn*L-UAA~*trn*L-UGU	spacer
57,000–57,500	17	LSC	*trn*Q-UUG~*acc*D	spacer
130,500–130,894	17	SSC	*ycf*1	coding
130,000–130,500	16	SSC	*ycf*2	coding
1500–2000	14	LSC	*psb*A~*trn*K-UUU; *trn*K-UUU	spacer; coding
25,000–25,500	14	LSC	*trn*G-UCC~*psb*Z	spacer
48,000–48,500	14	LSC	*atp*I~*atp*H	spacer
54,500–55,000	14	LSC	*trn*R-UCU~*trn*S-GCU	spacer
29,000–29,500	13	LSC	*psb*D; *psb*D~*trn*T-GGU	coding; spacer
29,000–29,500	13	LSC	*psb*D; *psb*D~*trn*T-GGU	coding; spacer
122,500–123,000	13	SSC	*ndh*A	coding
18,500–19,000	12	LSC	*ycf*3; *ycf*3~*psa*A	coding; spacer
48,500–49,000	12	LSC	*atp*I~*atp*H	spacer
125,500–126,000	12	SSC	*rps*15; *rps*15~ycf1	coding; spacer
0–500	11	LSC	*trn*H-GUG; *trn*H-GUG~*psb*A;*psb*A	coding; spacer; coding
13,000–13,500	11	LSC	*ndh*J~*trn*F-GAA; *trn*F-GAA*; trn*F-GAA~*trn*L-UAA	spacer; coding; spacer
4000–4500	10	LSC	*mat*K~*rbc*L	spacer
4500–5000	10	LSC	*mat*K~*rbc*L	spacer
31,500–32,000	10	LSC	*trn*E-UUC; *trn*E-UUC~*trn*Y-GUA; *trn*Y-GUA;*trn*Y-GUA~*trn*D-GUC	coding; spacer; coding; spacer
33,500–34,000	10	LSC	*psb*M~*pet*N; *pet*N; *pet*N~*trn*C-GCA	spacer; coding; spacer
49,500–50,000	10	LSC	*atp*H; *atp*H~*atp*F; *atp*F	coding; spacer; coding
58,000–58,500	10	LSC	*trn*U-UUG~*acc*D; *acc*D	spacer; coding
80,500–81,000	10	LSC	*rps*11; *rps*11~*rpl*36	coding; spacer
101,500–102,000	10	IRA	*rps*7~*trn*V-GAC	spacer
123,000–123,500	10	SSC	*ndh*A	coding

There were totally 311 insertions/deletions (indels) (0.20%) that had been found within the twelve cp genomes, and > 90% of which were short indels (1–10 bp) (Table 2). The distribution of these indels among the four cp genome regions was very similar to that of SNDs: LSC had the largest number (241, 0.27%), but SSC held the highest density (64, 0.34%), while the IR regions was the lowest in both number and density (6 per region, 0.02%) (Table 2, Fig. 2). The cp genome regions with the highest density of indels were found at two 500 bp-long sequence blocks: one included the intergenic spacer between the *trn*Q-UUG and *acc*D genes within LSC, while the other was within SSC and was composed of the intergenic spacer between the *rpl*32 and *trn*L-UAG genes, the *trn*L-UAG gene as well as the intergenic spacer between the *trn*L-UAG and *ccs*A genes. Within both regions, eleven indels were detected, respectively (Table 4). The result of the VISTA analysis is consistent with the results of SNDs and indels: almost the entire IR regions were conserved, while the identified conserved regions within SCs were short and scattered (Table 4).

**Table 4:** The genome areas identified to be rich in indels among the twelve acquired chloroplast genomes. Genome areas that have more than 5 indels per 500 bp are considered to be rich in indels

Genome position	Count	Region	Genomic context	Content
57,000–57,500	11	LSC	*trn*Q-UUG~*acc*D	spacer
115,500–116,000	11	SSC	*rpl*32~*trn*L-UAG; *trn*L-UAG; *trn*L-UAG~*ccs*A	spacer; coding; spacer
66,000–66,500	10	LSC	*psb*E~*pet*L	spacer
123,000–123,500	8	SSC	*ndh*A	coding
112,000–112,500	7	IRa;SSC	*trn*N-GUU~*ndh*F;*ndh*F	spacer; coding
53,000–53,500	6	LSC	*trn*R-UCU; *trn*R-UCU~*trn*S-GCU	coding; spacer
53,500–54,000	6	LSC	*trn*R-UCU~*trn*S-GCU	coding
56,000–56,500	6	LSC	*psb*K; *psb*K~*trn*Q-UUG	coding; spacer
10,000–10,500	5	LSC	*trn*V-UAC; *trn*V-UAC~*ndh*C	coding; spacer
13,000–13,500	5	LSC	*ndh*J~*trn*F-GAA; *trn*F-GAA; *trn*F-GAA~*trn*L-UAA	spacer; coding; spacer
29,500–30,000	5	LSC	*psb*D~*trn*F-GGU	spacer
69,000–69,500	5	LSC	*rps*18; *rps*18~*rps*20	coding; spacer
115,000–115,500	5	SSC	*rpl*32; *rpl*32~*trn*L-UAG	coding; spacer
119,500–120,000	5	SSC	*psa*C~*ndh*E; *ndh*E~*ndh*G	spacer; spacer

**Fig. 3:**
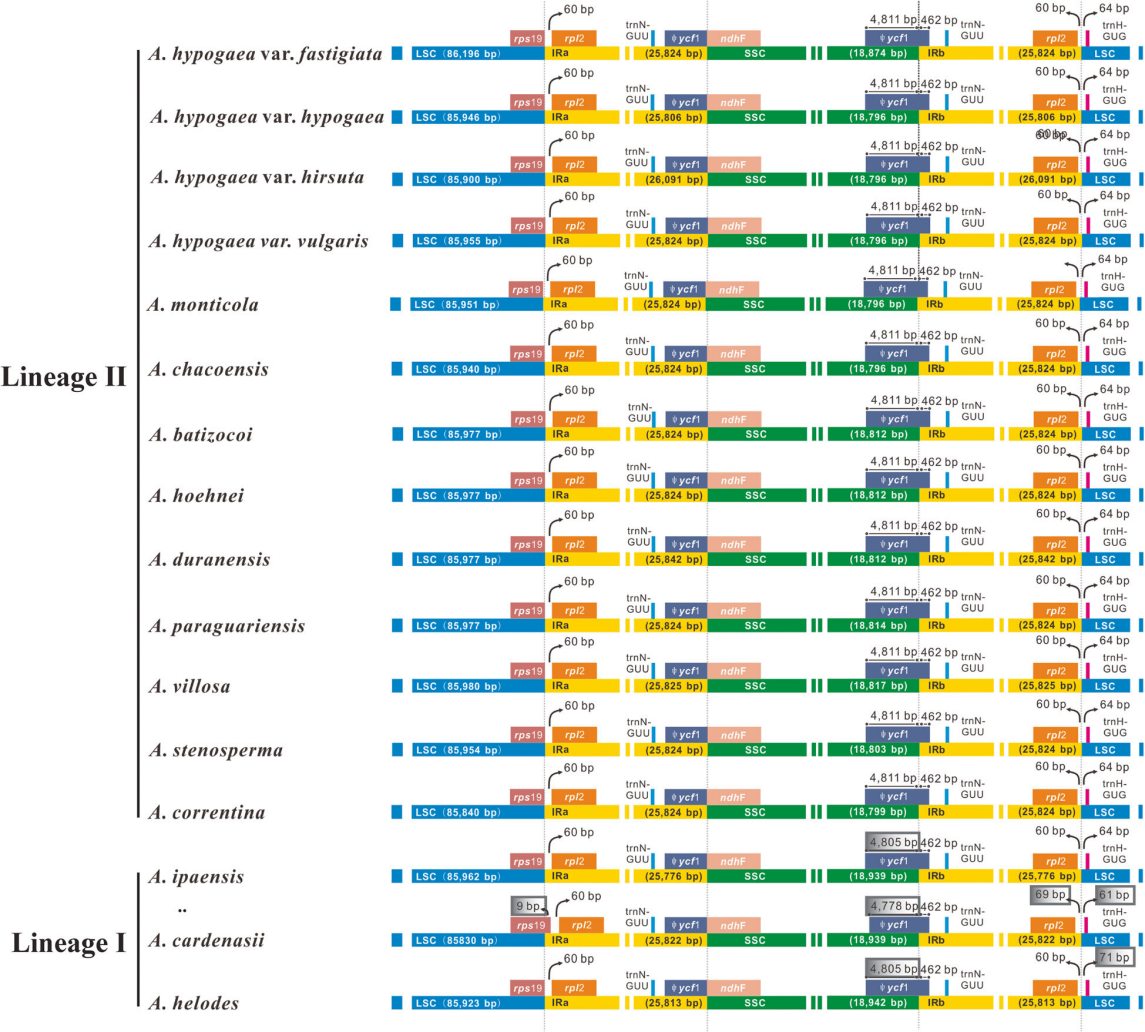
Detailed view of the IR-SC border regions among the twelve studied *Arachis* species. Regions that differ from the majority are highligted in grey boxes, which, according to our phylogenetic analyses, all belong to species within lineage I

Although the IR regions were rather conserved, the IR boundaries could vary greatly even within species [39], so in order to detect any possible IR border polymorphism, we compared the four IR-SC borders among the twelve assembled genomes, but no difference was found at the IRa-SSC border, while at the LSC-IRa, SSC-IRb and IRb-LSC borders, only small differences were discovered from *A*. *ipaënsis*, *A*. *cardenasii* and *A*. *helodes* (Fig. 3). The *rps*19 gene at the IRa-LSC boundary expanded 9 bp from the LSC region to the IRa side in *A*. *cardenasii* while it stops at the LSC-IRa junction in the rest of the species (Fig. 3). The length of the *ycf*1 gene (in SSC) at the SSC-IRb boundary was 4805 bp for *A*. *ipaënsis*/*A*. *helodes* and 4778 bp for *A*. *cardenasii*, which were shorter than those in the other species that were all 4811 bp (Fig. 3). On one side of the IRb-LSC boundary, the spacer between the *rpl*2 gene (in IRb) and the IRb-LSC junction was 69 bp long for *A*. *cardenasii* while this spacer for the rest of the species had a length of 60 bp. On the other side of the IRb-LSC boundary, the lengths of the spacers between the IRb-LSC junction and the *trn*H-GUG gene (in LSC) were, respectively, 61 bp and 71 bp for *A*. *cardenasii* and *A*. *helodes* while those of the rest species were all 64 bp (Fig. 3).

With MISA analysis, 69 universal SSR loci were detected within the twelve assembled cp genomes, among which 59 were mononucleotide, nine were dinucleotide and one was tetranucleotide. The majority of the identified SSRs were composed of A or T, which was consistent with earlier observations about cp SSRs in other taxa [40, 41]. 41 of the identified SSR loci showed variation among the twelve acquired *Arachis* cp genomes, and most of them (75.6%) were located in the LSC region, followed by SSC (24.4%), and none was found within IR (Additional file 3). PCR primers have been designed for each of the 41 variable SSR loci (Additional file 3).

### Phylogeny of the studied *Arachis* species

**Fig. 4:**
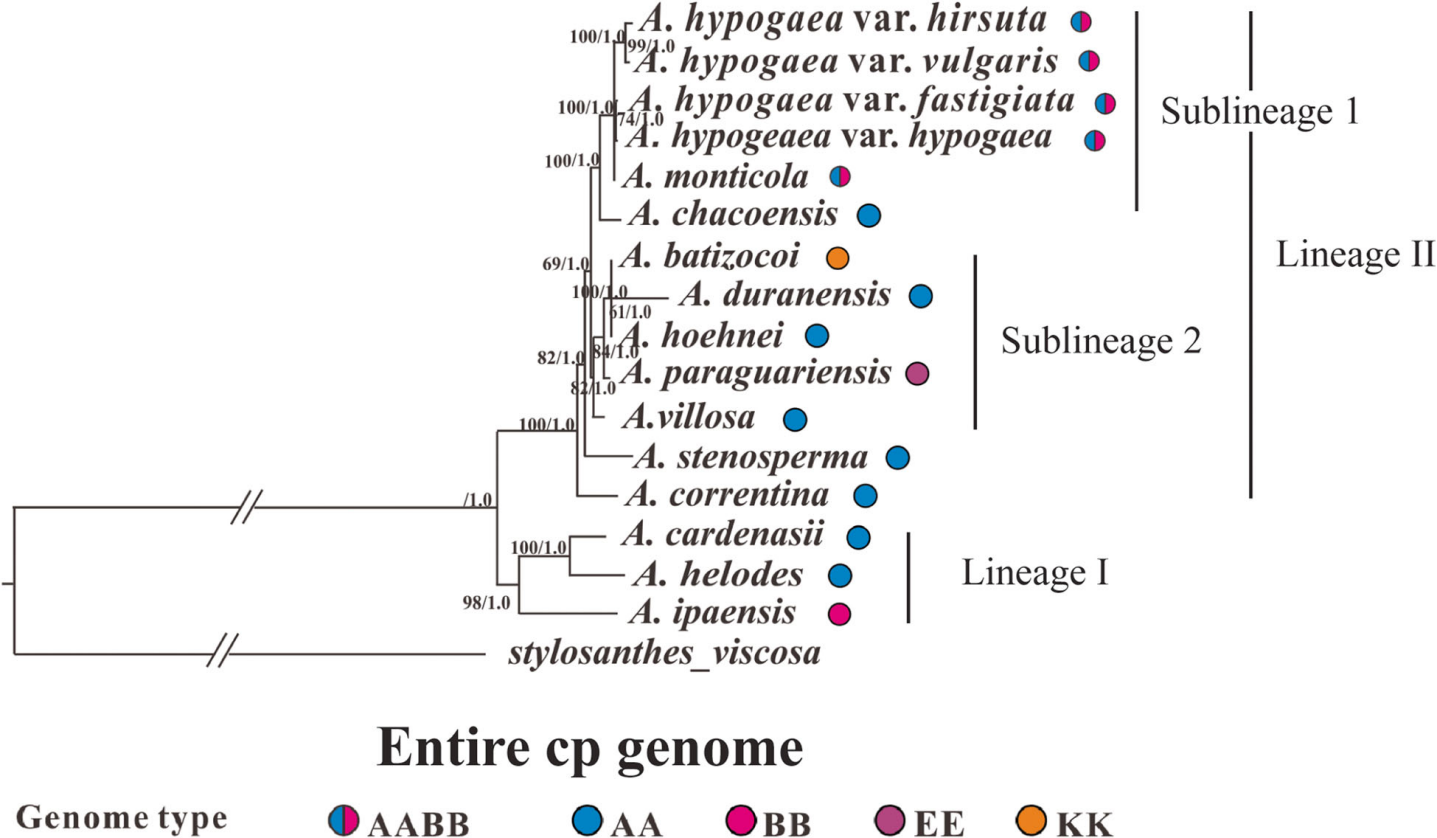
Bayesian inference tree for *Arachis* that is based on the entire cp genome. The tree is rooted with *Stylosanthes viscosa*. Two major lineages (I and II) have been observed among the Sect. *Arachis* species, and within lineage II, two sublineages (1 and 2) can also be recognized. For each node, the bootstrap support value is shown on the left and the Bayesian posterior probability is shown on the right. The genome type of each analyzed *Arachis* species are also given

**Fig. 5:**
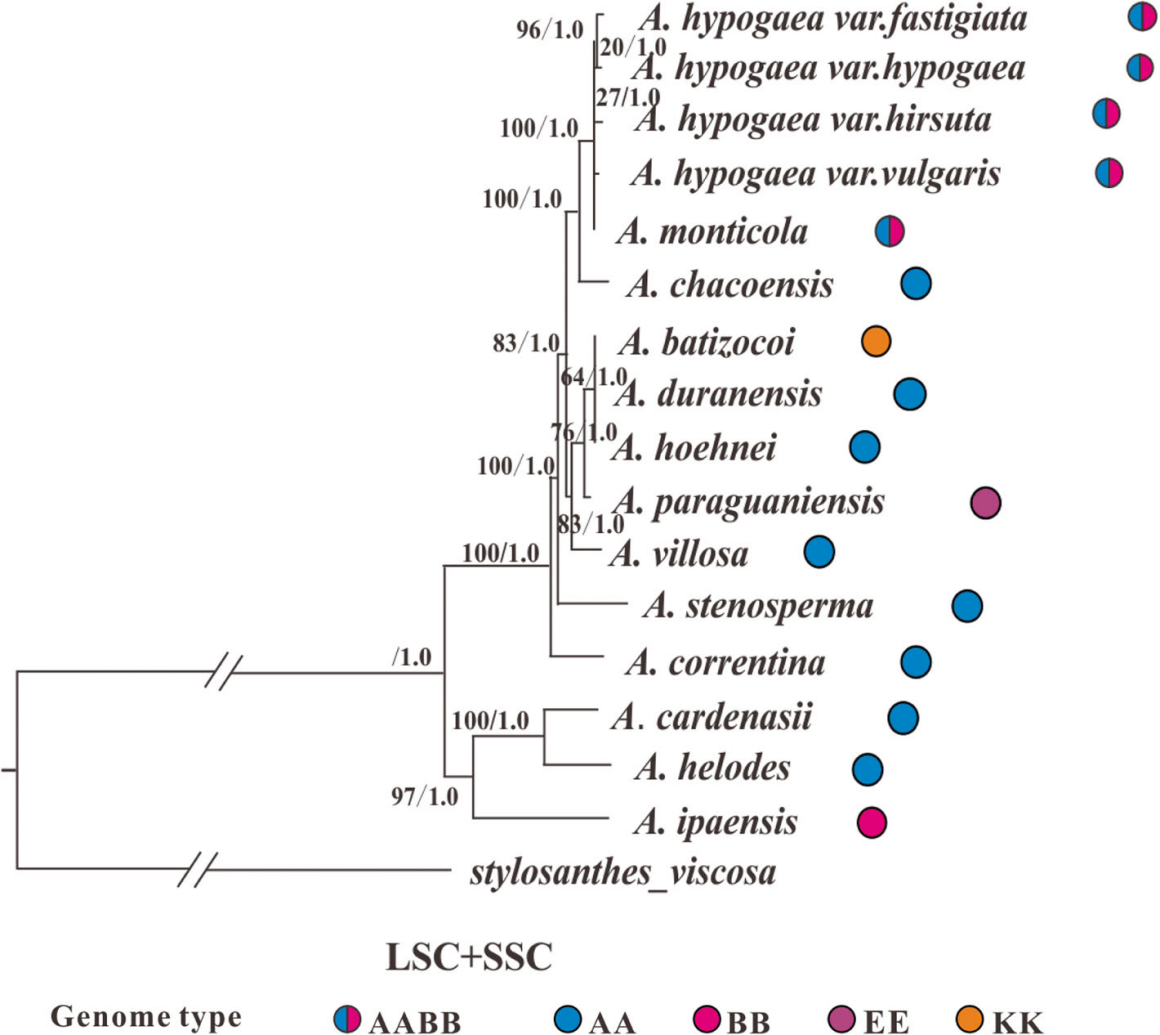
Bayesian inference tree for *Arachis* that is based on the two single copy genome regions. The tree is rooted with *Stylosanthes viscosa*. For each node, the bootstrap support value is shown on the left and the Bayesian posterior probability is shown on the right. The genome type of each analyzed *Arachis* species are also given. *A*. *chacoensis* is now known as *A*. *diogoi*

**Fig. 6:**
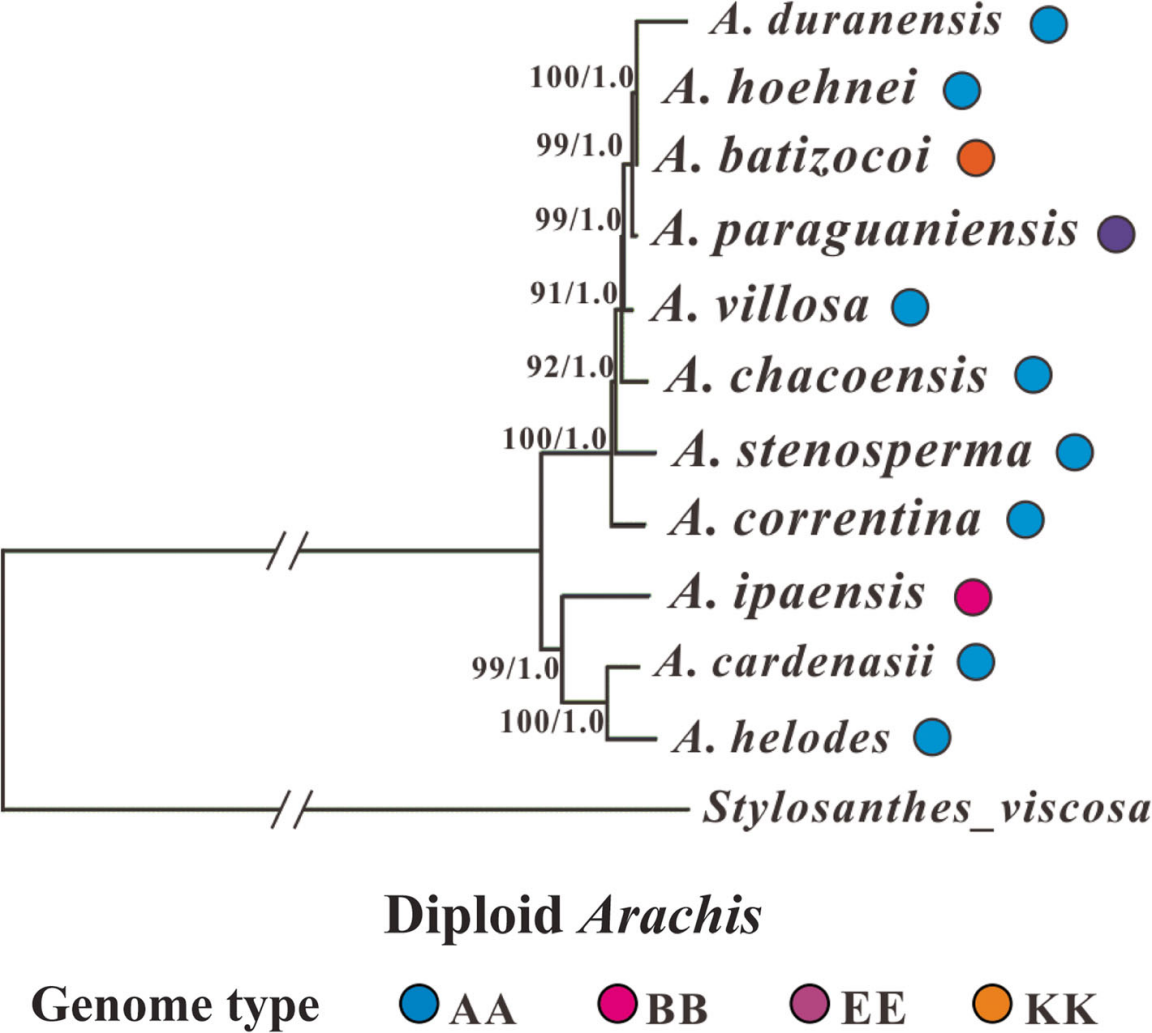
Bayesian inference tree for *Arachis* that only considers the diploid species. The tree is based on the entire cp genome and rooted with *Stylosanthes viscosa*. For each node, the bootstrap support value is shown on the left and the Bayesian posterior probability is shown on the right. The genome type of each analyzed *Arachis* species are also given. *A*. *chacoensis* is now known as *A*. *diogoi*

**Fig. 7:**
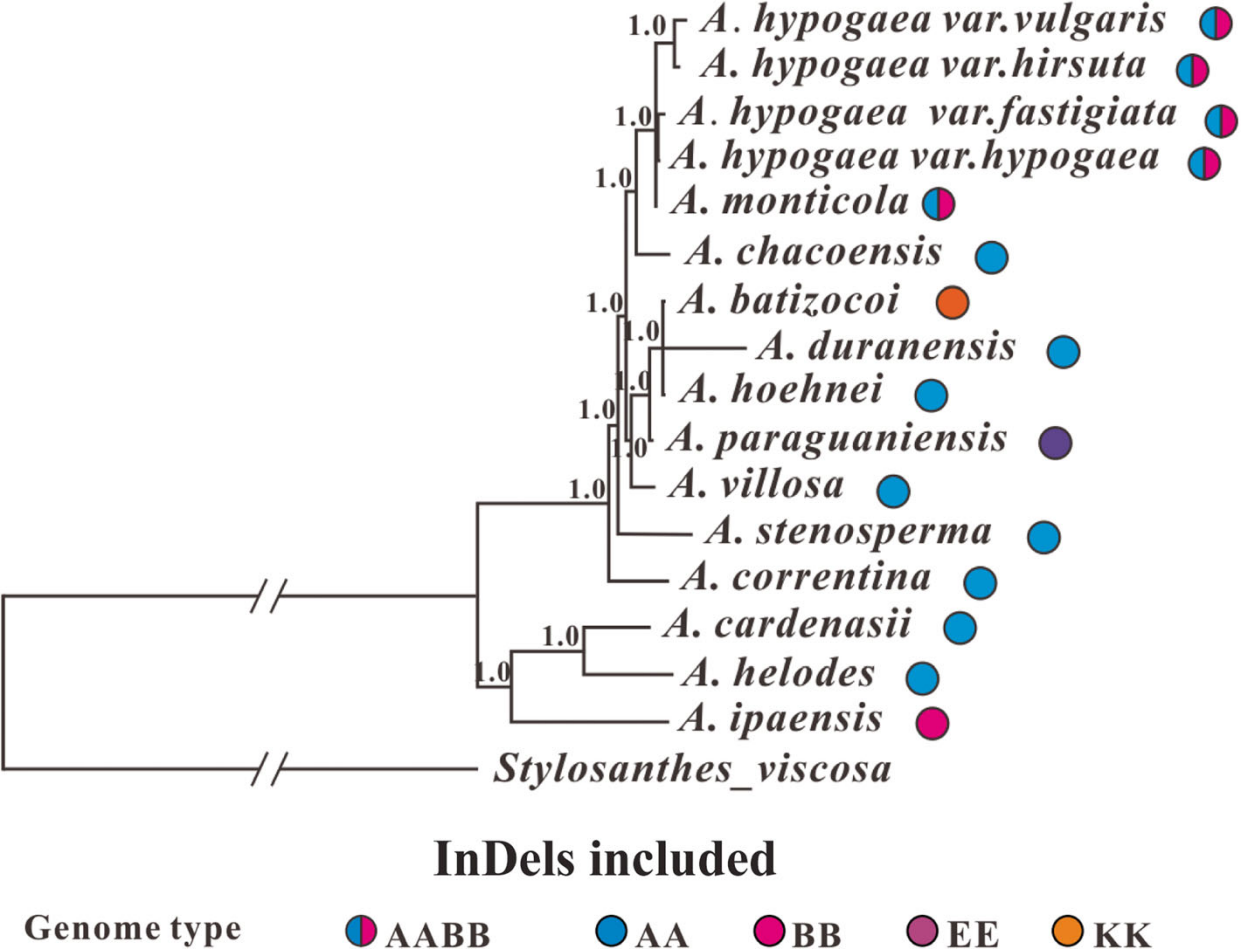
Bayesian inference tree for *Arachis* that also takes indels into consideration. The tree is based on the entire cp genome and rooted with *Stylosanthes viscosa*. For each node, the Bayesian posterior probability is shown. The genome type of each analyzed *Arachis* species are also given. *A*. *chacoensis* is now known as *A*. *diogoi*

Two different phylogenetic methods (Maximum Likelihood and Bayesian Inference) were used to infer the phylogenetic relationships between the analyzed species, and both methods generated nearly identical trees, we therefore only showed the Bayesian Inference phylogenetic tree (Fig. 4) (the Maximum Likelihood trees are available on request). Considering the much slower substitution rate of the IR regions that may not be suitable for inferring the relationship between closely related Sect. *Arachis* species, we therefore constructed phylogeny both with and without them. In addition, phylogenetic analyses of species differing in ploidy levels might produce unusual results comparing to those only involving species with the same ploidy level [1, 20], we thus tried to exclude the two tetraploid species (*A*. *hypogeae* and *A. monticola*) and only used the diploid species to infer phylogenetic trees with whole genome data. Furthermore, indels were not considered in the abovementioned phylogenetic analyses, however, information embedded within indels might help improve the resolution of phylogeny for closely related species [42]. We therefore performed Bayesian inferences of phylogeny that took indel information from the entire cp genome into consideration. However, among all the different situations we considered, the arrangement of major lineages and sublineages in the acquired phylogenetic trees always remained consistent (Figs. 4, 5, 6 and 7). Below, we would only present the results from the Bayesian Inference tree that was based on the whole cp genome data (excluding the indel information).

**Fig. 8:**
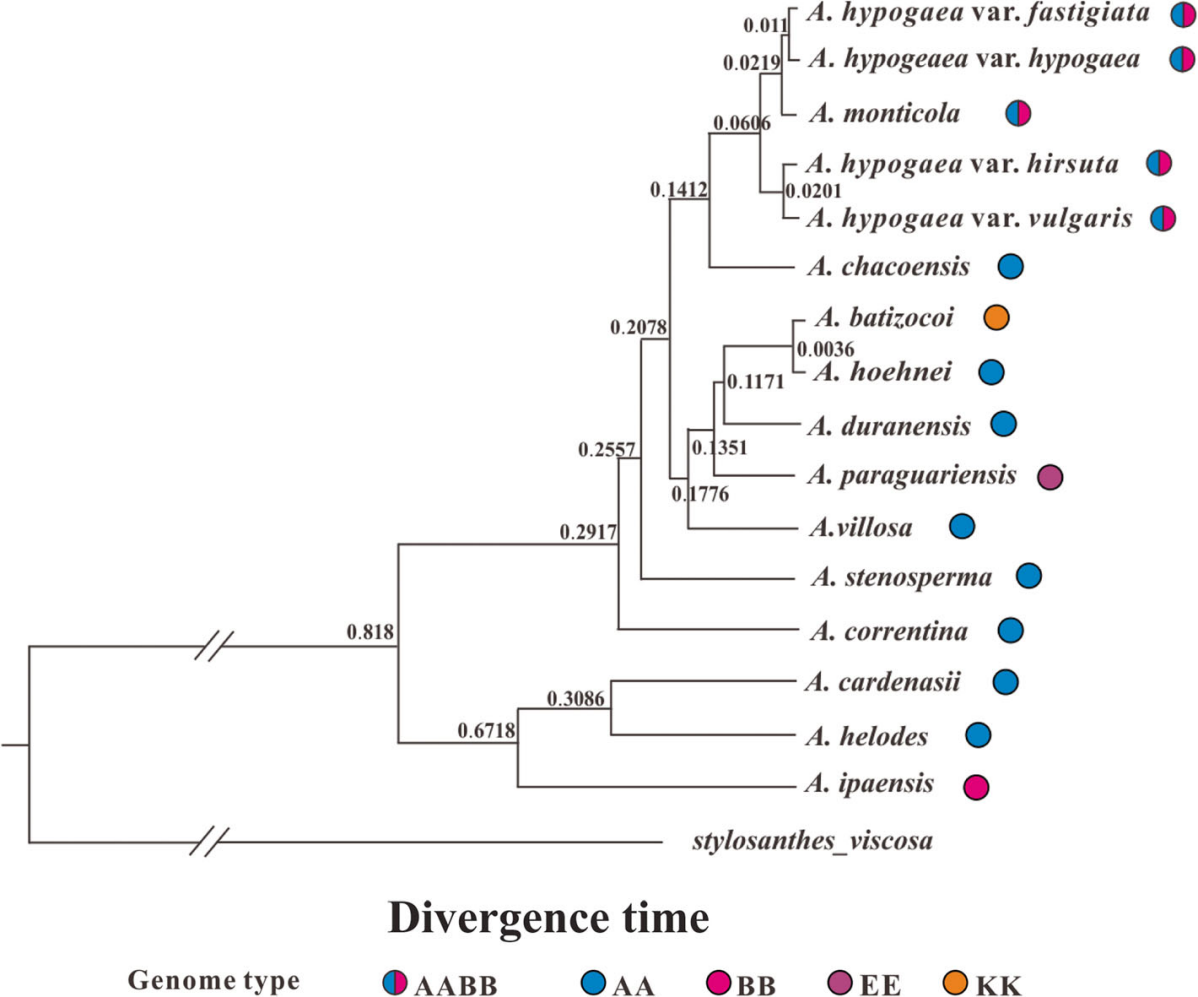
The estimated divergence time (Mya) among the analyzed *Arachis* taxa

A total of 16 *Arachis* genomes (the twelve genomes assembled in this study plus four earlier published *A. hypogaea* genomes) had been included in this part of the phylogenetic analyses, and they fell into two well-supported major lineages (I, II) in the inferred phylogenetic trees (Bootstrap [BS] value: 100; Bayesian posterior probability [BPP]: 1.0) (Fig. 4). Lineage I was composed of one BB genome species (*A*. *ipaënsis*) and two AA genome species (*A*. *cardenasii* and *A*. *helodes*), while lineage II comprised the rest of the species (AA genome species: *A*. *duranensis*, *A. hoehnei*, *A. chacoensis*, *A*. *villosa*, *A*. *stenosperma*, and *A. correntina*; AABB genome species: four *A. hypogaea* varieties and *A*. *monticola*; KK genome species: *A*. *batizocoi*; EE genome species: *A. paraguariensis*) (Fig. 4). Our molecular dating analysis implied that these two major lineages split 0.818 million years ago (Mya) (Fig. 8), and this divergence time was slightly shorter than the divergence time that was estimated between *A*. *duranensis* (in the present study belonging to lineage II) and *A*. *ipaënsis* (in the present study belonging to lineage I) (2.16 Mya) by Bertioli et al. [43], which was perhaps not surprising considering the latter was based on data from the bi-parentally inherited nuclear genome while the present study used cp genome (uni-parentally inherited) data. Both lineages didn’t differ much in their GC contents: 35.13% for lineage I while 35.09% for lineage II.

Within lineage I, the two AA genome species (*A*. *cardenasii* and *A*. *helodes*) grouped closely together (BS: 100; BPP: 1.0), and their most recent common ancestor was dated back to 0.3086 Mya (Figs. 4 and 8). In contrast, the split time between these two AA species and the BB genome species (*A*. *ipaënsis*) was much earlier: 0.6718 Mya (Fig. 8).

The time to the most recent common ancestor of lineage II was 0.2917, and the species within this lineage diverged rapidly (Fig. 8). Within lineage II, two distinct sublineages can be recognized: the first sublineage (BS: 100; BPP: 1.0) was composed of two AABB genome species (*A. hypogaea* and *A*. *monticola*) plus one AA genome species (*A. chacoensis*), and the most recent common ancestor of this sublineage was dated back to 0.1412 Mya (Figs. 4 and 8). Within this first sublineage, the two AABB genome species (the cultivated peanut and *A*. *monticola*) formed a highly supported clade (BS: 100; BPP: 1.0), the estimated time to the most recent common ancestor of this clade was 0.0606 Mya. The second sublineage (BS: 82; BPP: 1.0) within lineage II comprised three AA genome species (*A*. *duranensis*, *A. hoehnei*, *A*. *villosa*,), one KK (*A*. *bartizocoi*) and one EE genome species (*A. paraguariensis*). The split between these two sublineages might occur around 0.2078 Mya (Fig. 8).

## Discussion

The close wild relatives have been contributing valuable genetic resources to many economically important crops [44–47]. This study successfully acquired the complete cp genomes of twelve *Arachis* species that are closely related to the cultivated peanut (*A. hypogaea*), which is one of the most important oilseed crops worldwide. The rich genetic resources associated with the twelve *Arachis* cp genome data may hold great potential for future peanut cultivar improvement.

The same to those of other land plants [48–50], the *Arachis* cp genome is a single circular molecule that displays a quadripartite structure (LSC, IRa, SSC, IRb). Moreover, the genome size, gene composition and order, GC content, as well as IR-SC boundaries are also rather conserved among the *Arachis* cp genomes that have been acquired in this study (Additional file 1) and among thirteen earlier published *Arachis* cp genomes [21, 34–36]. Nevertheless, still substantial genetic variation (1368 SNDs and 311 indels) has been identified among the twelve acquired cp genomes, which, together with 69 SSR loci that have been detected in the present study, may serve as useful tools for future studies.

### The highly conserved IR regions

Among the four cp genome regions, the two IR regions are highly conserved comparing to the two SC regions, as reflected by the fact that less than 8% of the SNDs, indels and SSRs that have been identified in this study are located within the IR regions even though IRa and IRb constitute about one third of the genome (Fig. 1, Table 2). This low level of genetic variation at the IR regions (Comparing to SCs) is very common among plant species [51–53]. One possible explanation is that for the cp genome that exists as multiple copies within single plant cells, gene conversion with a tiny bias against new mutations would much more efficiently reduce the mutation load at the two IR (inverted repeat) regions than at the SC (single copy) regions due to the duplicative nature of the IRs [51, 52, 54–56]. Such conversion bias might arise from the base preference of the mismatch repair system during gene conversion [54, 57, 58].

### Two major lineages within sect. *Arachis*

There are two main genome types that have been identified within Sect. *Arachis*: AA (chromosome number: 2n = 20) and BB (2n = 20), which are also very important because the tetraploid cultivated peanut has a complicated genome (AABB; 4n = 40) that is composed of both of them [2]. Apart from AA, BB and AABB, Sect. *Arachis* species may also have a genome type of DD (2n = 20), FF (2n = 20), KK (2n = 20) or aneuploidy (2n = 18). Our phylogenetic analyses have shown that the analyzed Sect. *Arachis* taxa fall into two major lineages: the first one (lineage I) includes *A*. *ipaënsis*, which is the only BB genome species that has been studied here, as well as two AA genome species (Fig. 4). The second lineage (lineage II) comprises taxa that include two AABB genome species, six AA genome species, and one KK genome species. These two major lineages observed within Sect. *Arachis* are very well supported by bootstrap values (Fig. 4), if without the tetraploid *A. hypogaea* and *A*. *monticola* unifying them; they may be well distinguished as separate taxonomic sections [1, 20]. Our divergence time estimation shows that the most recent common ancestor of these two major lineages is dated back to 0.818 Mya, and from this common ancestor lineage I was first derived (at least 0.6718 Mya), while lineage II was derived rather recently (0.2917 Mya) and rapidly (Fig. 8). The cp genome size and structure, the gene content and order, and the GC content are well conserved between the two lineages. However, the IR-SC border regions of the lineage I species cp genomes have been found to vary in length and differ from those of the lineage II species that are all identical (Fig. 3). The presence of two major Sect. *Arachis* lineages is confirmed by nuclear genome data generated by genotyping-by-sequencing (GBS) approach (unpublished observations).

Similar species grouping (into two major lineages) within Sect. *Arachis* has also been observed in a number of other studies (Additional file 4) [11, 14, 20–22, 24, 25, 59–62]. These studies may work on different *Arachis* species, and use very different methods, genetic markers or sequence data, but lineage II or the equivalent, in almost all of these studies, is dominated by the AA genome species and tends to exclude genome types other than AA and AABB (Additional file 4). In contrast, lineage I or the equivalent is more diverse and can take on any genome type (AA, BB, AABB, DD, FF, KK or aneuploidy) that has been identified within Sect. *Arachis* but slightly prefers the BB genome species (Additional file 4).

Interestingly, the AA genome species within Sect. *Arachis* are all perennial (except *A*. *duranensis*, *A*. *stenosperma* and *A. hoehnei*), while all the other various genome types (e.g. BB, DD, FF, KK and AABB) belong to annuals/biennial species (Sect. *Arachis* is one of the only two *Arachis* sections that have annual species; the other one is Sect. *Heteranthae*) [4]. The genome type compositions of annual/biennial and perennial species may be consistent with the earlier finding that annual species generally have a much higher molecular evolutionary rate than perennial species [63–65]. Moreover, in the place of origin for *Arachis*, South America, the distribution areas of annual/biennial species have relatively more diverse environmental conditions than those of perennial species. In South America, the overall geographic distribution of Sect. *Arachis* species has a bizarre shape that is kind of similar to a capital T [4]. The perennial Setc. *Arachis* species prevail the vertical axis of this T shaped range, which more or less coincides with the meridians 57° and 58° west and mainly includes the watersheds of the Paraguay and Uruguay rivers [4]. Whereas the annual/biennial species dominate the two arms of the “T” shaped geographic distribution: the Tocantins river to the east, the Mamoré river and the Guaporé river to the west, as well as the dry “charco” region (up to the foothills of the Andes) to the southwest [4]. At these arm regions, the annual/biennial species are usually adapted to very stressful environmental conditions, such as prolonged inundation and periodic drought [4].

Although the presence of two major lineages within Sect. *Arachis* and the overall pattern of the species component of these lineages are evident, however, to which lineage one Sect. *Arachis* species should be placed is not always clear. For example, according to the present study, the KK genome species, *A*. *batizocoi*, falls with most of the analyzed AA genome species into lineage II, however, *A*. *batizocoi* has been found to appear in lineage I-equivalent clade by three earlier chloroplast phylogenetic studies [14, 20, 21]. Another example is that the AA genome species *A*. *cardenasii* and *A*. *helodes*, which fall into lineage I at the present study, have, however, often been found in lineage II-equivalent clade by earlier studies [11, 20, 21, 60]. Moreover, *A. paraguariensis* (belonging to Sect. *Erectoide*), which is the only non-Sect. *Arachis* species acquired by the present study, was originally chosen as an outgroup but turned out to be closely related to *A*. *batizocoi* (KK), *A. hoehnei* (AA) and *A*. *duranensis* (AA) within lineage II (Fig. 4). It is worth noting that similar species mixing between *Arachis* sections are not uncommon [11, 20, 21, 24, 66]. For example, Yin et al. [21] studied seven *Arachis* species, five of which belong to Sect. *Arachis* while the rest two are the members of Sect. *Procumbentes*; instead of grouping together, the two Sect. *Procumbentes* species were, respectively, nested within different Sect. *Arachis* species groups.

These incongruences of species relationship that are mentioned above may be the result of several different reasons. First, a high level of genetic variation has been reported within different *Arachis* species (especially the wild ones) [4–10, 67]. This high level of intraspecific genetic variation may be at least partly due to the autogamous reproductive system and the underground fruiting habit of the *Arachis* species, which can seriously restrain interspecific and intraspecific gene flows [4]. Therefore, different samples of the same species may have distinct genetic constitution (such as *A*. *duranensis*, see below) and phylogeny inference based on these different samples is then likely to result in very different species relationship, for this reason, more representative samples from each species need to be considered for future phylogenetic study. Second, *Arachis* species are not always completely incompatible with each other, hybridization even between different genome types or sections have been well documented [2, 14, 15, 59]; the interspecific hybrids that are possibly produced in nature may well blur the species boundaries. Third, considering the rather recent divergence of the Sect. *Arachis* species (< 1 Mya, Fig. 8), ancestral polymorphism is also likely to be maintained within extant species and consequently complicate phylogeny inference. In addition, differences in analyzing method (such as UPGMA and maximum likelihood), in genetic data type (AFLP, RFLP and DNA sequence etc.), in the amount of genetic information that is considered (single genes or genome data), as well as in the inheritance mode (bi-parental or uni-parental) of the acquired data set will all have an impact on the inference of species relationship [29].

### The maternal origin of the cultivated peanut (*A*. *hypogeae*)

Between the two major Sect. *Arachis* lineages that have been observed from the phylogenetic trees inferred by the present study, it is lineage II that the cultivated peanut falls into, and within this group, the four considered *A*. *hypogeae* varieties mix together with *A*. *monticola*. This result is not in conflict with previous views that *A*. *monticola* may be the direct wild ancestor of the cultivated peanut [11] or as an introgressive derivative between the cultivated peanut and wild *Arachis* species [68, 69]. These earlier views are based on a combination of different evidences. For example, these two species are the only tetraploid species within Sect. *Arachis* and they usually group together in phylogenetic analyses [2, 26, 66]. Moreover, the geographic distributions of these two species are close to each other [70] and hybridization between them has been well documented [14, 16].

From the discussion above, we have already known that lineage II or the equivalent is dominated by AA genome species and particularly “disfavors” BB genome species, and this is especially true for the phylogenetic trees that are inferred from cp sequences as reflected by the present and three earlier studies [14, 20, 21]. Actually in all of these four studies, the cultivated peanut is nested within lineage II or the equivalent. Considering the chloroplast genome is maternally inherited in *Arachis* as shown by an earlier study, where the F1 hybrids between two *Arachis* species always grouped together with their maternal parents in the phylogenetic tree that was built based on chloroplast DNA [14], our result suggests that the maternal genome donor to the tetraploid cultivated peanut (AABB) and the tetraploid wild peanut species (*A*. *monticola*; AABB) is one AA genome species [14, 20, 71], or one can say that the A genome of *A*. *hypogeae* and *A*. *monticola* is contributed by their maternal progenitor.

Currently, the most popular view regarding exactly which species serve as the genome donors to *A*. *hypogeae* and *A*. *monticola* is that *A*. *duranensis* (AA) and *A*. *ipaënsis* (BB) contribute, respectively, the A and B genomes. This generally accepted opinion is supported by evidences from genome type, geographic distribution, crossability, cytogenetic analysis, molecular analysis, phylogenetic analysis and genome sequence comparison [11, 14, 43, 60, 70–72]. However, in the present study, *A*. *hypogeae* and *A*. *monticola* group closely with neither *A*. *duranensis* nor *A*. *ipaënsis* in the inferred phylogenetic tree (Fig. 4), instead, these two tetraploid species form a well-supported subgroup with *A. chacoensis* (AA). Our result alone, however, cannot conclude that *A. chacoensis*, instead of *A*. *duranensis*, serves as the A genome donor to *A*. *hypogeae* and *A*. *monticola* due to a combination of different reasons. First of all, *A*. *duranensis* has a relatively wide geographical distribution and lots of intraspecific variation has been reported within this species [8, 22, 71, 72], if different samples that have distinct genetic makeups are used for phylogenetic inference, the result may be very different. Next, *A*. *duranensis* has been shown to be able to hybridize with other *Arachis* species [14], leading to interspecific gene flow that may also blur the species boundary. At last, although chloroplast genomes have a lot of advantages in phylogenetic analysis as mentioned in Introduction, they are however relatively vulnerable to problems like introgression and the retention of ancestral polymorphism that are frequently encountered when inferring the phylogenetic relationship between closely related species, due to their maternal inheritance mode [73, 74].

## Conclusions

The highly variable wild peanut species may serve as a rich source of useful alleles for the improvement of the cultivated peanut, which is one of the most important oilseed crops in the world. The present study has acquired the complete cp genome sequences of twelve *Arachis* species and eleven of which belong to Sect. *Arachis*; the cultivated peanut is also a member of Sect. *Arachis*. As for other land plant species [48–50], the cp genome size and structure, as well as gene content and order are highly conserved among the twelve acquired cp genomes. Nevertheless, substantial SNDs, indels and SSRs have been identified from the acquired genomes, and most of these SNDs, indels, and SSRs are distributed at the two single copy genome regions (LSC and SSC). The two inverted repeat genome regions (IRa and IRb) have a very low level of genetic variation, which may be due to biased gene conversions. Phylogenetic analyses of the acquired genomes have identified two major lineages (I and II) within Sect. *Arachis*. Our results together with many earlier studies show that lineage II is dominated by AA genome species that are mostly perennial while the genome types of the lineage I species are rather diverse; Sect. *Arachis* species with genome types other than AA are all annual/biennial [4]. In addition, the tetraploid cultivated peanut is found within lineage II, which together with the maternal inheritance mode of chloroplast suggest that it is an AA genome species that served as the maternal donor of the cultivated peanut. In summary, the twelve cp genomes acquired in the present study have not only helped us understand the genetic basis and phylogenetic relationships of the *Arachis* species better, but also provided us with substantial genetic resources that may be valuable for future peanut improvement.

## Methods

### Plant material and genome sequencing

A total of twelve *Arachis* species that belong to Sect. *Arachis* (*A. monticola*, *A. duranensis*, *A. stenosperma*, *A. batizocoi*, *A. cardenasii*, *A. helodes*, *A. correntina*, *A. hoehnei*, *A. chacoensis* (now known as *A. diogoi*) and *A. villosa*, *A. ipaënsis*) and Sect. *Erectoides* (*A. paraguariensis ssp. paraguariensis*) were included in the present study (Table 1). The seeds for these wild peanut species (except *A. ipaënsis*, see below) originally came from, and were identified as well by, the National Wild *Arachis* Nursery, Oil Crop Research Institute, Chinese Academy of Agricultural Sciences, Wuhan, China. All the analyzed species have an AA genome type, except *A. batizocoi* (KK), *A. ipaënsis* (BB), and *A. paraguariensis* (EE) [2]. Leaves from single one-month-old plants representing each of these species (except *A. ipaënsis*) (grown under greenhouse conditions) were collected for total genomic DNA isolation using the DNeasy Plant Mini Kit (QIAGEN, Germany). DNA quality was checked by agarose gel electrophoresis using Super GelRed dye (US Everbright Inc., Suzhou, China). A single paired-end library with an average insert size of about 350 bp was constructed for all analyzed samples (different species are “indexed” with different barcoded adapters) following the manufacturer’s protocol (Illumina, Beijing, China). The constructed library first had its quality assessed on Caliper LabChip GX using the High Sensitivity Assay Kit (Caliber, USA), and was then hybridized and amplified on a flow cell to generate clonal clusters on the cBOT platform using the Truseq PE Cluster Kit v3-cBot-HS (Illumina, Beijing, China). Whole-cp genome sequencing by synthesis was performed on the Illumina Hiseq Xten platform using the Truseq v3-HS kit (Illumina, Beijing, China).

### Genome assembly and annotation

The Illumina sequencing generated > 1 Gb raw paired-end reads for each sample, and these data were deposited into NCBI Sequence Read Archive (SRA) (BioProject Accession No. PRJNA543570). These raw paired-end reads, plus one raw read dataset for *A. ipaënsis* that was downloaded from the NCBI SRA database (accession number: SRX2701518) [37], were analyzed using the NGS QC ToolKit v2.3.3 for filtering low quality data after quality check and removing adaptor sequences [75]; the cut-off values for the percentage of read length and phred score were set, respectively, to 80 and 30. In total, 305,336–3,503,151 high-quality reads were acquired per sample, which produced 293–3362 fold cp genome coverage when being mapped onto a reference cp genome from *A. hypogaea* (GenBank [76] accession number KX257487, [35]) using bowtie [77] (Additional file 1). The high-quality reads (obtained from last step) that belong to the cp genome were extracted and assembled into contigs using the de novo assembler SPAdes v3.9.0 [78] (with several different k-mer sizes: 93, 105, 117 and 121). These assembled contigs were further assembled into complete cp genomes by NOVOPlasty v2.6.2, which has been designed specifically for assembling organelle genomes [79]. The complete cp genome was annotated using the DOGMA tool with default parameters [80]. OGDraw v1.2 [81] was used to draw complete cp genome images.

For each of the assembled complete cp genomes, with the help of the sequin software [82], a SQN file was generated. The SQN file was then submitted to GenBank (accession numbers: MK144818 for *A. monticola*, MK144823 for *A. paraguariensis*, MK144822 for *A. duranensis*, MK144819 for *A. stenosperma*, MK144820 for *A. batizocoi*, MK144824 for *A. cardenasii*, MK144826 for *A. helodes*, MK144828 for *A. correntina*, MK144827 for *A. hoehnei*, MK144821 for *A. chacoensis* (now known as *A. diogoi*), MK144829 for *A. ipaënsis* and MK144825 for *A. villosa*).

### Genetic variation analysis

Both SNDs and indels were detected from the mapping (to the *A. hypogaea* reference genome) results of the bowtie analyses (see above) using GATK [83] (ploidy setting = 1). The VISTA server [84] was used to identify the conserved genome regions. The visualization of the densities of SNDs and indels (i.e. the number of SNDs or indels counted for every consecutive 500 bp blocks), and the conserved regions over the entire cp genome were performed using Circos [85]. Simple sequence repeats (SSRs) were predicted using MISA [86] with default parameters except the minimum counts of the repeat unit within single SSR motifs: (10) mono-, (6) di-, (5) tri-, (4) tetra-, (3) penta-, and (3) hexa-nucleotide repeat units [21]. All the identified SSRs were manually checked and redundant results were removed.

### Phylogenetic analysis and divergence time estimation

To better understand the species relationships within *Arachis*, especially within Sect. *Arachis*, phylogenetic analyses were performed on the complete cp genome data. Apart from the twelve cp genomes that were acquired by the present study, four earlier published cp genomes from different *A*. *hypogeae* botanical varieties (GenBank accession no. MG814006 for var. *fastigiata* Waldron, MG814007 for var. *hirsute* Kohler, MG814008 for var. *hypogaea* L., MG814009 for var. *vulgaris* Harz) [36] were also included in the analyses, so, in total 16 complete cp genomes from thirteen *Arachis* species were considered for the phylogenetic inference. In addition, a cp genome of *Stylosanthes viscosa* L. (GenBank accession no. MG735675) was chosen as an outgroup for the phylogenetic analysis; this cp genome showed the highest similarity to *Arachis* species [87] among the cp genomes that were available up to the date when the analysis was performed. Before being used for the phylogenetic analyses, the 17 cp genomes were aligned with the HolmBlocks pipeline (with default settings unless specified) [88], which was fast and efficient especially for handling a large amount of divergent interspecies sequence data and was therefore suitable for overcoming the alignment difficulties that may be introduced by the relatively distantly related outgroup species. Within the HolmBlocks pipeline, progressiveMauve [89] was first used to identify conserved genome regions, based on which a preliminary alignment was next constructed; the alignment was then trimmed by Gblocks [90] to remove poorly aligned and divergent regions.

The phylogenetic analyses were first carried out using the Maximum Likelihood methods as implemented in IQ-TREE [91]. Ten independent searches were performed, and the statistical confidence in each predicted node was evaluated with 10,000 non-parametric bootstrap replicates. MrBayes v3.2.5 [92] was then used to perform Bayesian inferences of phylogeny via Markov Chain Monte Carlo method [92]. We run the inferences for 100,000 Markov Chain Monte Carlo generations, with a sampling frequency of 1000 generations. Results of the first 25% generations were discarded as burn-ins and the rest were used to build a 50% majority-rule consensus tree.

Land plant cp genomes were characterized by four typical regions: two IR regions and two SC regions, (see Results) [38]. The IR regions were shown to have a much lower nucleotide substitution rate comparing with the SC regions [52, 54, 93], so might not be suitable for inferring the phylogeny of the closely related Sect. *Arachis* species as analyzed by the present study. Here, we therefore reconstructed the phylogeny using the same methods as above but excluding the IR regions.

Phylogenetic analyses of species differing in ploidy levels might produce unusual results comparing to those only involving species with the same ploidy level [1, 20], in order to test whether this is the case with our study, we then excluded the two tetraploid species (*A*. *hypogeae* and *A. monticola*) and only used the diploid species for inferring phylogenetic trees with whole genome data.

Moreover, indels were not considered in the abovementioned phylogenetic analyses, however, information that was embedded within indels might help improve the resolution of phylogeny for recently divergent species [42]. We therefore performed the last phylogenetic analysis (for this study) that took the 311 indels observed in our *Arachis* whole-genome data into consideration. In this step, the indels were first converted into binary data with the simple indel coding method [94] using the SeqState software [95]. The acquired binary data together with the ordinary nucleotide substitution information were input into MrBayes as mixed data for Bayesian inferences of *Arachis* phylogeny.

At last, the software BEAST v1.7.2 [96] was used to estimate the divergence time among different *Arachis* species. The estimated divergence time between genus *Stylosanthes* and *Arachis* from Saslis-Lagoudakis et al. [97] was used as a calibration point.

## Supplementary information

**Additional file 1. **Basic characteristics of the twelve *Arachis* genomes that have been acquired in this study. *A. chacoensis* is now known as *A. diogoi*.

**Additional file 2.** The genes that have been identified from the twelve acquired chloroplast genomes. Intron-containing genes are marked by asterisks (*).

**Additional file 3. **The SSR makers that have been developed from the twelve acquired *Arachis* chloroplast genomes.

**Additional file 4. **A summary of earlier studies that have identified two major Sect. *Arachis* lineages.

## Data Availability

The plant samples that are used in the present study are available from the corresponding author on reasonable request. The datasets that have been generated (assembled complete chloroplast genome accession numbers: MK144818 for *Arachis monticola*, MK144823 for *A. paraguariensis*, MK144822 for *A. duranensis*, MK144819 for *A. stenosperma*, MK144820 for *A. batizocoi*, MK144824 for *A. cardenasii*, MK144826 for *A. helodes*, MK144828 for *A. correntina*, MK144827 for *A. hoehnei*, MK144821 for *A. chacoensis*, MK144829 for *A. ipaënsis* and MK144825 for *A. villosa*) or analyzed (raw reads of chloroplast genome: SRX2701518 for *A. ipaënsis*; cultivated peanut chloroplast genomes: KX257487, MG814006, MG814007, MG814008, MG814009) for this study can be found in GenBank (https://www.ncbi.nlm.nih.gov/genbank). The raw data supporting the conclusions of this manuscript, the Maximum Likelihood trees built in this study, the phylogenetic tree based on nuclear genome data generated by genotyping-by-sequencing (GBS) approach (unpublished observations) are available from the corresponding author on reasonable request. All the other datasets for this study are included in the manuscript and the additional files.

## Abbreviations

BPP: Bayesian posterior probability
BS: bootstrap
cp: chloroplast
indel: insertion/deletion
IR: inverted repeat (region)
IRa, IRb: two IR regions that are identical but in opposite orientations
LSC: long single copy (region)
Mya: million years ago
SC: single copy (region)
SND: single nucleotide divergence
SSC: short single copy (region)
