# Supplementary material for: Twelve complete chloroplast genomes of wild peanuts: great genetic resources and a better understanding of Arachis phylogeny
Source: BMC Plant Biol. 2019 Nov 19;19:504. doi: 10.1186/s12870-019-2121-3 (PMC6862822; doi:10.1186/s12870-019-2121-3)
Supplement: Supplementary file 1 — Additional file 1. Basic characteristics of the twelve Arachis genomes that have been acquired in this study. A. chacoensis is now known as A. diogoi. [file 12870_2019_2121_MOESM1_ESM.docx]

**Additional file 1.** Basic characteristics of the twelve *Arachis* genomes that have been acquired in this study. *A*. *chacoensis* is now known as A. diogoi.

|  | *A. paraguariensis* | *A. duranensis* | | *A. monticola* | | *A. stenosperma* | *A. batizocoi* | *A. cardenasii* | *A.*  *helodes* | *A. correntina* | *A. hoehnei* | *A. chacoensis* | *A.*  *villosa* | *A. ipaënsis* |
| --- | --- | --- | --- | --- | --- | --- | --- | --- | --- | --- | --- | --- | --- | --- |
| Raw reads | 902,050 | | 1,096,682 | | 1,975,032 | 3,503,151 | 1,540,720 | 1,549,786 | 1,729,390 | 305,336 | 1,451,528 | 1,226,360 | 1,034,358 | 557,870 |
| Genome size (bp) | 156,437 | | 156,413 | | 156,384 | 156,287 | 156,473 | 156,491 | 156,437 | 156,395 | 156,437 | 156,405 | 156,447 | 156,453 |
| Mean coverage (×) | 864.93 | | 1051.71 | | 1894.4 | 3362.22 | 1476.98 | 1485.5 | 1658.22 | 292.85 | 1391.8 | 1176.13 | 991.73 | 534.86 |
| LSC length (bp) | 85,977 | | 85,830 | | 85,940 | 85,840 | 85,977 | 85,923 | 85,977 | 85,951 | 85,977 | 85,954 | 85,980 | 85,962 |
| SSC length (bp) | 18,812 | | 18,939 | | 18,796 | 18,799 | 18,812 | 18,942 | 18,812 | 18,796 | 18,814 | 18,803 | 18,817 | 18,939 |
| IR length (bp) | 25,824 | | 25,822 | | 25,824 | 25,824 | 25,842 | 25,813 | 25,824 | 25,824 | 25,824 | 25,824 | 25,825 | 25,776 |
| LSC GC content (%) | 33.8 | | 33.8 | | 33.8 | 33.8 | 33.8 | 33.8 | 33.8 | 33.8 | 33.8 | 33.8 | 33.8 | 33.8 |
| SSC GC content (%) | 30.3 | | 30.2 | | 30.2 | 30.2 | 30.3 | 30.2 | 30.3 | 30.3 | 30.3 | 30.3 | 30.3 | 29.9 |
| IR GC content (%) | 42.9 | | 42.9 | | 42.9 | 42.9 | 43.0 | 42.9 | 42.9 | 42.9 | 42.9 | 42.9 | 42.9 | 42.9 |
| GC content (%) | 36.3 | | 36.4 | | 36.3 | 36.3 | 36.3 | 36.3 | 36.3 | 36.3 | 36.3 | 36.3 | 36.3 | 36.3 |
| Total | 110 | | 110 | | 110 | 110 | 110 | 110 | 110 | 110 | 110 | 110 | 110 | 110 |
| Protein coding genes | 76 | | 76 | | 76 | 76 | 76 | 76 | 76 | 76 | 76 | 76 | 76 | 76 |
| rRNA genes | 4 | | 4 | | 4 | 4 | 4 | 4 | 4 | 4 | 4 | 4 | 4 | 4 |
| tRNA genes | 30 | | 30 | | 30 | 30 | 30 | 30 | 30 | 30 | 30 | 30 | 30 | 30 |
