# Supplementary material for: Twelve complete chloroplast genomes of wild peanuts: great genetic resources and a better understanding of Arachis phylogeny
Source: BMC Plant Biol. 2019 Nov 19;19:504. doi: 10.1186/s12870-019-2121-3 (PMC6862822; doi:10.1186/s12870-019-2121-3)
Supplement: Supplementary file 2 — Additional file 2. The genes that have been identified from the twelve acquired chloroplast genomes. Intron-containing genes are marked by asterisks (*). [file 12870_2019_2121_MOESM2_ESM.docx]

**Additional file 2.** The genes that have been identified from the twelve acquired chloroplast genomes. Intron-containing genes are marked by asterisks (*).

| **Gene categories** | **Gene groups** | **Gene names** |
| --- | --- | --- |
| Self-replication | rRNA genes | *rrn5, rrn4.5, rrn16, rrn23* |
|  | tRNA genes | **trnA-UGC, trnC-GCA, trnD-GUC, trnE-UUC, trnF-GAA, trnG-GCC, *trnG-UCC, trnH-GUG, trnI-CAU, *trnI-GAU,*trnK-UUU, trnL-CAA, *trnL-UAA, trnL-UAG, trnfM-CAU,trnM-CAU, trnN-GUU, trnP-UGG, trnQ-UUG,trnR-ACG, trnR-UCU, trnS-GCU, trnS-GGA, trnS-UGA, trnT-GGU,trnT-UGU, trnV-GAC, *trnV-UAC, trnW-CCA, trnY-GUA* |
|  | small subunit of ribosome | *rps2, rps3, rps4, rps7, rps8, rps11, *rps12, rps14,rps15, *rps16, rps18, rps19* |
|  | large subunit of ribosome | *rpl2, rpl14, *rpl16, rpl20, rpl22, rpl23, rpl32, rpl33,rpl36* |
|  | DNA dependent RNA polymerase | *rpoA, rpoB, *rpoC1, rpoC2* |
| Genes for photosynthesis | Subunits of NADH-dehydrogenase | **ndhA, *ndhB, ndhC, ndhD, ndhE, ndhF,ndhG, ndhH, ndhI, ndhJ, ndhK* |
|  | Subunits of photosystem I | *psaA, psaB, psaC, psaI, psaJ* |
|  | Subunits of photosystem II | *psbA, psbB, psbC, psbD, psbE, psbF, psbH, psbI, psbJ, psbK, psbL, psbN, psbT, psbZ* |
|  | Subunits of cytochrome b/f complex | *petA, *petB, *petD, petG, petL, petN,* |
|  | Subunits of ATP synthase | *atpA, atpB, atpE, *atpF, atpH, atpI* |
|  | Large subunit of rubisco | *rbcL* |
|  | Assembly/stability of photosystems I | **ycf3*, *ycf4* |
| Other genes | Maturase | *matK* |
|  | Protease | **clpP* |
|  | carbon metabolism | *cemA* |
|  | Subunit of Acetyl-CoA-carboxylase | *accD* |
|  | c-type cytochrome synthesis gene | *ccsA* |
| Genes of unknown function | Open Reading Frames (ORF, ycf) | *ycf1*, *ycf2* |
