# Supplementary material for: Twelve complete chloroplast genomes of wild peanuts: great genetic resources and a better understanding of Arachis phylogeny
Source: BMC Plant Biol. 2019 Nov 19;19:504. doi: 10.1186/s12870-019-2121-3 (PMC6862822; doi:10.1186/s12870-019-2121-3)
Supplement: Supplementary file 3 — Additional file 3. The SSR makers that have been developed from the twelve acquired Arachis chloroplast genomes. [file 12870_2019_2121_MOESM3_ESM.docx]

**Additional file 3.** The SSR makers that have been developed from the twelve acquired *Arachis* chloroplast genomes.

| **NO** | **Genomic context** | **Start** | **End** | **Content** | **SSR type** | **Region** | **Forward sequence** | **Reverse sequence** | **Length** |
| --- | --- | --- | --- | --- | --- | --- | --- | --- | --- |
| 1 | *mat*K-*rbc*L | 4362 | 4371 | spacer | (A)10 | LSC | CTACCGAGGTTATGGACGAA | AAGGATACCACTCTTGACAAAG | 189 |
| 2 | *mat*K-*rbc*L | 4677 | 4686 | spacer | (A)10 | LSC | AATTGCCTCTGTAACGTGAAGA | GGATTGAGCCGAAGACAAAGAT | 489 |
| 3 | *rbc*L-*atp*B | 6793 | 6802 | spacer | (A)10 | LSC | GAATTATCGCAACAACCAGA | TTCACCATAGCGGCTTAC | 460 |
| 4 | *rbc*L-*atp*B | 6877 | 6887 | spacer | (A)11 | LSC | TCTAGCATTGGGTAGACCTCAT | CATCGGCACCTATTGCTCATAT | 213 |
| 5 | *ndh*C-*ndh*J | 11016 | 11025 | spacer | (A)10 | LSC | CGTGCTTATCCTAATTGTTGGT | CCATAGAGAAGCGGCCATAA | 226 |
| 6 | *ycf*3 | 17866 | 17875 | intron | (A)10 | LSC | GTCATTACGTGCGACTATCTCC | TACGGCGCTTCCTCTATCAAT | 198 |
| 7 | *ycf*3-*psa*A | 18650 | 18660 | spacer | (T)11 | LSC | TGACTGCTGAGCCGTATGA | TGGATGGACTGATGTAGACAAC | 482 |
| 8 | *ycf*3-*psa*A | 19512 | 19521 | spacer | (T)10 | LSC | CCGGATCATAATTGTTCTAGTG | CGGCGAACGAATAATCATTG | 186 |
| 9 | *psb*D-*trn*T-GGU | 29461 | 29470 | spacer | (A)10 | LSC | AAGGAAGTCTGAGATGTATGGA | CTTTCTCTTACCGTGATCTACA | 692 |
| 10 | *psb*D~*trn*T-UGU | 29704 | 29723 | spacer | (TATC)5 | LSC | AGATCACGGTAAGAGAAAGAGA | AATGACTGGGAGAAAGAGACTT | 190 |
| 11 | *trn*M-CAU-*trn*E-UUC | 31062 | 31073 | spacer | (A)12 | LSC | CGAATTCTGGTTATCCTCATCA | TATCTCTGTCTCATCCTAAGCC | 229 |
| 12 | *trn*M-CAU -*trn*E-UUC | 31337 | 31346 | spacer | (G)10 | LSC | AGATTGGCGATTGGAATGAACA | TCCTGGACCACTAGACGATG | 211 |
| 13 | *rpo*C1 | 41278 | 41292 | coding | (A)15 | LSC | CCGTACCGACTCAAGATATGC | AAGAAATCGTGAGGGTTCAAGT | 196 |
| 14 | *rp*oC1 | 41317 | 41328 | coding | (A)12 | LSC | CGGATTGGCTGTCTTGTGTT | CGATAGGAGCTTCTCTTGAGGA | 369 |
| 15 | *atp*I-*atp*H | 48695 | 48705 | spacer | (T)11 | LSC | AGATTCAGTTCTTCGGTCGAA | GAAATTGACAGTAGCAGGACAA | 369 |
| 16 | *trn*R-UCU *trn*S-GCU | 53565 | 53574 | spacer | (A)10 | LSC | AGAAGACCTCTGTCCTATCCAT | ATTCCCGCTACCCGCTTT | 393 |
| 17 | *trn*R-UCU -*trn*S-GCU | 54098 | 54107 | spacer | (A)10 | LSC | GCTATTGTGCCTCAACCTCTC | CCCGCCCTCCTTTGATTCA | 328 |
| 18 | *psb*K~*trn*Q-UUG | 56563 | 56576 | spacer | (A)14 | LSC | ACTCTACTCGAATCCATTTGTG | GTTGTTCGTCAATTCCATCTCA | 294 |
| 19 | *trn*Q-UUG -accD | 57140 | 57150 | spacer | (T)11 | LSC | TCCACATCAATCGTCAAACTCT | CCGCAAATTCCTTCGATATTGT | 197 |
| 20 | *trn*Q-UUG *acc*D | 57776 | 57786 | spacer | (A)11 | LSC | CTTCGTGGGAACCCTTATCTAA | TGGTATTTACTTGGTCCAGGAT | 204 |
| 21 | *pe*tA-*psb*J | 63824 | 63834 | spacer | (T)11 | LSC | TGCAGTACTAGTCAGTCATAGT | GCTCAAAGAAGGGAATCAACAA | 354 |
| 22 | *rp*s12-*clp*P | 71049 | 71059 | spacer | (A)11 | LSC | TTTGTTCCATTTCCGACGAGAT | TGTATGGCTTGTATCCGAAGAC | 285 |
| 23 | *clp*P | 71622 | 71636 | intron | (T)15 | LSC | TACTATGATGGTTCCGTTGCTT | GCCTATTTCAGTGTCACAAACT | 177 |
| 24 | *clp*P | 71649 | 71662 | intron | (AT)7 | LSC | ATACTATGATGGTTCCGTTGCT | GAAGCCTATTTCAGTGTCACAA | 181 |
| 25 | *clp*P | 72398 | 72407 | intron | (T)10 | LSC | CTAACGAAGGGAAAGGAAAGGT | AGAAGCCCATTCAGAAACAAGA | 200 |
| 26 | *clp*P~*psb*B | 73550 | 73559 | spacer | (A)10 | LSC | CCACACCACCATTGCGTATT | CCAGCAACCAGAGCCGTAT | 336 |
| 27 | *rp*s11-*rp*136 | 80968 | 80979 | spacer | (TA)6 | LSC | AAGGAAGTCTGAGATGTATGGA | TCTCTTTCTCTTACCGTGATCT | 695 |
| 28 | *rp*s11-*rp*136 | 80993 | 81004 | spacer | (TA)6 | LSC | ACATCGGTAACAGTGACAA | AGCCAGTTTCCTAGACATAA | 728 |
| 29 | *rp*s11-*rp*136 | 81196 | 81205 | spacer | (T)10 | LSC | CCTACAAGAGCCAATCGTTCT | TTGATCCGTAGACGAGGTAGAA | 293 |
| 30 | *rp*116-*rp*s3 | 84217 | 84234 | spacer | (AT)9 | LSC | TTATTGACTCGGTGCTCTTGAT | TCGCTTCGCATTATCTGGATT | 336 |
| 31 | *rp*116-*rp*s3 | 84400 | 84414 | spacer | (T)15 | LSC | TTATTGACTCGGTGCTCTTGAT | AACTCATCGCTTCGCATTATCT | 342 |
| 32 | *ndh*F-*rp*132 | 114439 | 114448 | spacer | (A)10 | SSC | ATAGGAACTGGAAGCGGAATG | TTCGATTGGGTCATGTCATATG | 408 |
| 33 | *rp*132 | 114993 | 115002 | coding | (A)10 | SSC | TATATCAGTTGCATGGCAGTTC | CCAGCTAATTCAGAGTATTCCA | 194 |
| 34 | *rp*132-*trn*L-UAA | 115676 | 115685 | spacer | (A)10 | SSC | TCATATGTATGGCGCAACC | CTAAGAGCAGCGTGTCTAC | 308 |
| 35 | *rp*132-*trn*L-UAA | 115691 | 115701 | spacer | (A)11 | SSC | CATATGTATGGCGCAACC | GGCTTACCCGAAATCATAA | 273 |
| 36 | *ndh*E-*ndh*G | 119949 | 119958 | spacer | (A)10 | SSC | GCATCATTGACGAACTCCT | GGCTCGCCAGTAAGAAAT | 251 |
| 37 | *ndh*E-*ndh*G | 120033 | 120044 | spacer | (T)12 | SSC | GACGAACTCCTTATCAATTTCG | TCATCGTCGAGAATGGAATAGT | 381 |
| 38 | *ndh*A | 122751 | 122761 | intron | (A)11 | SSC | GGCATATCGGTTGAAGTGGAT | CATGAGTGATTCGGCGGATT | 198 |
| 39 | *ndh*A | 123295 | 123306 | intron | (TA)6 | SSC | CGTGGTGGAAGTCATCAGTTC | GCTTCATATTCGCCTGGAGTT | 179 |
| 40 | *ycf*1 | 128753 | 128765 | coding | (T)13 | SSC | CTATGGAAATATGCCCTCTTGT | AGTTGGTTGTATTCGCCAAT | 281 |
| 41 | *ycf*1 | 129268 | 129278 | coding | (A)11 | SSC | CAAATATCCTTGATCGTGAGAC | CATTCCGCTTCCAGTTCCT | 334 |
