# Supplementary material for: Twelve complete chloroplast genomes of wild peanuts: great genetic resources and a better understanding of Arachis phylogeny
Source: BMC Plant Biol. 2019 Nov 19;19:504. doi: 10.1186/s12870-019-2121-3 (PMC6862822; doi:10.1186/s12870-019-2121-3)
Supplement: Supplementary file 4 — Additional file 4. A summary of earlier studies that have identified two major Sect. Arachis lineages. [file 12870_2019_2121_MOESM4_ESM.docx]

**Additional file 4.** A summary of earlier studies that have identified two major Sect. *Arachis* lineages. The number of species, the genome type and the species belonging to the AA genome type are highlighted in bold.

|  | **Number of species/genome type (species name)** | |  |
| --- | --- | --- | --- |
| **References** | **Lineage II** | **Lineage I** | **Data type** |
| Gimenes et al., 2002a [1] | **5/AA (*A*. *correntina*, *A*. *helodes*, *A*. *cardenasii*, *A*. *duranensis*, *A*. *kempff-mercadoi*)**, **1/BB** (*A*. *ipaënsis*), **1/AABB** (*A*. *hypogaea*) | **1/BB** (*A*. *magna*), **1/KK** (*A*. *batizocoi*) | RFLP |
| Jung et al., 2003 [2] | **2/AA (*A*. *cardenasii*, *A*. *duranensis*)**, **2/AABB** (*A*. *hypogaea*, *A*. *monticola*) | **1/BB** (*A*. *ipaënsis*), **2/AABB** (*A*. *hypogaea*, *A*. *monticola*) | Stearoyl-ACP desaturase gene sequence |
| Jung et al., 2003 [2] | **2/AA (*A*. *cardenasii*, *A*. *duranensis*),** **2/AABB** (*A*. *hypogaea*, *A*. *monticola*) | **1/BB** (*A*. *ipaënsis*), **2/AABB** (*A*. *hypogaea*, *A*. *monticola*) | Oleoyl-PC desaturase gene sequence |
| Tallury et al., 2005 [3] | **5/AA (*A*. *duranensis*, *A*. *kuhlmannii*, *A*. *hoehnei*, *A*. *kempff-mercadoi*, *A*. *herzogii*)**, **1/DD** (*A*. *glandulifera*), **1/FF** (*A*. *benensis*), **2/KK** (*A*. *cruziana*, *A*. *batizocoi*), **2/2n=18** (*A*. *palustris*, *A*. *praecox*) | **2/BB** (*A*. *williamsii*, *A*. *ipaënsis*), **1/AABB** (*A*. *hypogaea*) | AFLP |
| Tallury et al., 2005 [3] | **5/AA (*A*. *duranensis*, *A*. *kuhlmannii*, *A*. *hoehnei*, *A*. *kempff-mercadoi*, *A*. *herzogii*)**, **1/AABB** (*A*. *hypogaea*) | **2/BB** (*A*. *williamsii*, *A*. *ipaënsis*), **1/DD** (*A*. *glandulifera*), **1/FF** (*A*. *benensis*), **2/KK** (*A*. *cruziana*, *A*. *batizocoi*), **2/2n=18** (*A*. *palustris*, *A*. *praecox*) | *trn*T-F (chloroplast) |
| da Cunha et al., 2008 [4] | **6/AA (*A*. *hoehnei*, *A*. *schininii*, *A*. *kuhlmannii*, *A*. *cardenasii*, *A*. *microsperma*, *A*. *simpsonii*)** | **1/AA** (*A*. *duranensis*), **2/BB** (*A*. *gregoryi*, *A*. *magna*), **2/AABB** (*A*. *hypogaea*, *A*. *monticola*), **1/KK** (*A*. *batizocoi*) | RAPD |
| Bechara et al., 2010 [5] | **12/AA (*A*. *simpsonii*, *A*. *hoehnei*, *A*. *correntina*, *A*. *villosa*, *A*. *cardenasii*, *A*. *duranensis*, *A*. *schininii*, *A*. *microsperman*, *A*. *stenosperma*, *A*. *kuhlmannii*, *A*. *linearifolia*, *A*. *helodes*)**, **2/AABB** (*A*. *hypogaea*, *A*. *monticola*) | **4/BB** (*A*. *williamsii*, *A*. *ipaënsis*, *A*. *valida*, *A*. *magna*), **1/DD** (*A*. *glandulifera*), **2/KK** (*A*. *cruziana*, *A*. *batizocoi*), **3/2n=18** (*A*. *decora*, *A*. *palustris*, *A*. *praecox*) | ITS (1&2), 5.8s rDNA |
| Friend et al., 2010 [6] | **8/AA (*A*. *correntina*, *A*. *duranensis*, *A*. *herzogii*, *A*. *kempff-mercadoi*, *A*. *kuhlmannii*, *A*. *helodes*, *A*. *diogoi*, *A*. *hoehnei*)**, **2/AABB** (*A*. *hypogaea*, *A*. *monticola*) | **4/BB** (*A*. *valida*, *A*. *magna*, *A*. *ipaënsis* *A*. *williamsii*), **2/AABB** (*A*. *hypogaea*, *A*. *monticola*), **1/DD** (*A*. *glandulifera*), **2/FF** (*A*. *trinitensis*, *A*. *benensis*), **2/KK** (*A*. *cruziana*, *A*. *batizocoi*), **2/2n=18** (*A*. *palustris*, *A*. *praecox*) | ITS |
| Friend et al., 2010 [6] | **8/AA (*A*. *correntina*, *A*. *duranensis*, *A*. *herzogii*, *A*. *kempff-mercadoi*, *A*. *kuhlmannii*, *A*. *helodes*, *A*. *diogoi*, *A*. *hoehnei*)**, **2/AABB** (*A*. *hypogaea*, *A*. *monticola*) | **4/BB** (*A*. *valida*, *A*. *magna*, *A*. *ipaënsis*, *A*. *williamsii*), **1/DD** (*A*. *glandulifera*), **2/FF** (*A*. *trinitensis*, *A*. *benensis*), **2/KK** (*A*. *cruziana*, *A*. *batizocoi*), **2/2n=18** (*A*. *palustris*, *A*. *praecox*) | *trn*T-F (chloroplast) |
| Wang et al., 2011 [7] | **14/AA (*A*. *hoehnei*, *A*. *diogoi*, *A*. *correntina*, *A*. *villosa*, *A*. *cardenasii*, *A*. *simpsonii*, *A*. *duranensis*, *A*. *schininii*, *A*. *microsperma*, *A*. *kempff-mercadoi*, *A*. *stenosperma*, *A*. *kuhlmannii*, *A*. *linearifolia*, *A*. *helodes*)**, **2/AABB** (*A*. *hypogaea*, *A*. *monticola*) | **4/BB** (*A*. *williamsii*, *A*. *ipaënsis*, *A*. *valida*, *A*. *magna*), **1/DD** (*A*. *glandulifera*), **2/KK** (*A*. *cruziana*, *A*. *batizocoi*), **3/2n=18** (*A*. *decora*, *A*. *palustris*, *A*. *praecox*) | ITS |
| Moretzsohn et al., 2012 [8] | **14/AA (*A*. *cardenasii*, *A*. *schininii*, *A*. *hoehnei*, *A*. *duranensis*, *A*. *kuhlmannii*, *A*. *microsperman*, *A*. *simpsonii*, *A*. *stenosperma*, *A*. *villosa*, *A*. *correntina*, *A*. *kempff-mercadoi*, *A*. *diogoi*, *A*. *helodes*, *A*. *linearifolia*)**, **1/BB** (*A*. *valida*), **2/AABB** (*A*. *hypogaea*, *A*. *monticola*), **1/DD** (*A*. *glandulifera*), **3/KK** (*A*. *cruziana*, *A*. *krapovickasii*, *A*. *batizocoi*) | **4/BB** (*A*. *williamsii*, *A*. *gregoryi*, *A*. *magna*, *A*. *ipaënsis*), **2/AABB** (*A*. *hypogaea*, *A*. *monticola*), **3/2n=18** (*A*. *decora*, *A*. *palustris*, *A*. *praecox*) | 3 introns |
| Moretzsohn et al., 2012 [8] | **14/AA (*A*. *cardenasii*, *A*. *diogoi*, *A*. *linearifolia*, *A*. *kuhlmannii*, *A*. *helodes*, *A*. *kuhlmannii*, *A*. *stenosperma*, *A*. *kempff-mercadoi*, *A*. *microsperman*, A. simpsonii, *A*. *duranensis*, *A*. *villosa*, *A*. *correntina*, *A*. *hoehnei*), 1/BB** (*A*. *valida*), **1/DD** (*A*. *glandulifera*), **1/FF** (*A*. *benensis*), **3/KK** (*A*. *cruziana*, *A*. *krapovickasii*, *A*. *batizocoi*), **3/2n=18** (*A*. *decora*, *A*. *palustris*, *A*. *praecox*) | **1/AA** (*A*. *schininii*), **5/BB** (*A*. *gregoryi*, *A*. *magna*, *A*. *williamsii*, *A*. *ipaënsis*, *A*. *valida*) | SSRs |
| He et al., 2014 [9] | **3/AA (*A*. *diogoi*, *A*. *duranensis*, *A*. *stenosperma*)** | **1/BB** (*A*. *ipaënsis*), **1/DD** (*A*. *glandulifera*) | 7 gene-related sequences |
| Leal-Bertioli et al., 2014 [10] | **2/AA (*A*. *stenosperma*, *A*. *duranensis*)** | **2/BB** (*A*. *ipaënsis*, *A*. *magna*), **1/KK** (*A*. *batizocoi*) | 16 intron sequences |
| Yin et al., 2017 [11] | **3/AA (*A*. *helodes*, *A*. *diogoi*, *A*. *villosa*)**, **1/AABB** (*A*. *hypogaea*) | **1/KK** (*A*. *batizocoi*) | Chloroplast genome |
| The present study | **6/AA (*A*. *correntina*, *A*. *stenosperma*, *A*. *villosa*, *A*. *duranensis*, *A*. *hoehnei*, *A*. *chacoensis* [= *A*. *diogoi*])**, **2/AABB** (*A*. *hypogaea*, *A*. *monticola*), **1/KK** (*A*. *batizocoi*) | **2/AA** (*A*. *cardenasii*, *A*. *helodes*), **1/BB** (*A*. *ipaënsis*) | Chloroplast genome |

**References**

1. Gimenes MA, Lopes CR, Galgaro ML, Valls JFM, Kochert G. RFLP analysis of genetic variation in species of section *Arachis*, genus *Arachis* (Leguminosae). Euphytica*.* 2002;123:421-9.

2. Jung S, Tate PL, Horn R, Kochert G, Moore K, Abbott AG. The phylogenetic relationship of possible progenitors of the cultivated peanut. J Hered*.* 2003;94:334-40.

3. Tallury SP, Hilu KW, Milla SR, Friend SA, Alsaghir M, Stalker HT, Quandt D. Genomic affinities in *Arachis* section *Arachis* (Fabaceae): molecular and cytogenetic evidence. Theor Appl Genet*.* 2005;111:1229-37.

4. da Cunha FB, Nobile PM, Hoshino AA, de Carvalho Moretzsohn M, Lopes CR, Gimenes MA. Genetic relationships among *Arachis hypogaea* L. (AABB) and diploid *Arachis* species with AA and BB genomes. Genet Resour Crop Evol*.* 2008;55:15-20.

5. Bechara MD, Moretzsohn MC, Palmieri DA, Monteiro JP, Bacci Jr M, Martins Jr J, Valls JF, Lopes CR, Gimenes MA. Phylogenetic relationships in genus *Arachis* based on ITS and 5.8 S rDNA sequences. BMC Plant Biol*.* 2010;10:255.

6. Friend SA, Quandt D, Tallury SP, Stalker HT, Hilu KW. Species, genomes, and section relationships in the genus *Arachis* (Fabaceae): a molecular phylogeny. Plant Syst Evol*.* 2010;290:185-99.

7. Wang CT, Wang XZ, Tang YY, Chen DX, Cui FG, Zhang JC, Yu SL. Phylogeny of *Arachis* based on internal transcribed spacer sequences. Genet Resour Crop Evol*.* 2011;58:311-9.

8. Moretzsohn MC, Gouvea EG, Inglis PW, Leal-Bertioli SCM, Valls JFM, Bertioli DJ. A study of the relationships of cultivated peanut (*Arachis hypogaea*) and its most closely related wild species using intron sequences and microsatellite markers. Ann Bot*.* 2012;111:113-26.

9. He GH, Barkley NA, Zhao YL, Yuan M, Prakash CS. Phylogenetic relationships of species of genus *Arachis* based on genic sequences. Genome*.* 2014;57:327-34.

10. Leal-Bertioli SCM, Santos SP, Dantas KM, Inglis PW, Nielen S, Araujo ACG, Silva JP, Cavalcante U, Guimaraes PM, Brasileiro ACM. Arachis batizocoi: a study of its relationship to cultivated peanut (A. hypogaea) and its potential for introgression of wild genes into the peanut crop using induced allotetraploids. Ann Bot*.* 2014;115:237-49.

11. Yin DM, Wang Y, Zhang XG, Ma XL, He XY, Zhang JH. Development of chloroplast genome resources for peanut (*Arachis hypogaea* L.) and other species of *Arachis*. Sci Rep*.* 2017;7:11649.
